# Genome-wide mosaicism within *Mycobacterium abscessus*: evolutionary and epidemiological implications

**DOI:** 10.1186/s12864-016-2448-1

**Published:** 2016-02-17

**Authors:** Guillaume Sapriel, Julie Konjek, Mickael Orgeur, Laurent Bouri, Lise Frézal, Anne-Laure Roux, Emilie Dumas, Roland Brosch, Christiane Bouchier, Sylvain Brisse, Mathias Vandenbogaert, Jean-Michel Thiberge, Valérie Caro, Yun Fong Ngeow, Joon Liang Tan, Jean-Louis Herrmann, Jean-Louis Gaillard, Beate Heym, Thierry Wirth

**Affiliations:** EA3647-EPIM, UFR des Sciences de La Santé, Université de Versailles St. Quentin, Montigny le Bretonneux, France; Laboratoire des Sciences du Climat et de l’Environnement, LSCE, UMR8212, Université de Versailles St. Quentin - CEA - CNRS, Saint-Aubin, France; Atelier de Bioinformatique, ISYEB, UMR 7205, Paris, France; AP-HP, Hôpital Ambroise Paré, Service de Microbiologie et Hygiène, Boulogne-Billancourt, France; Institut Pasteur, Unit for Integrated Mycobacterial Pathogenomics, Paris, France; Institut of Biology of the Ecole Normale Supérieure, 46 rue d’Ulm, 75230 Paris, Cedex 05 France; PF1-Plate-Forme Génomique, Institut Pasteur, Paris, France; Institut Pasteur, Genotyping of Pathogens and Public Health, Paris, France; Department of Medical Microbiology, Faculty of Medicine, University of Malaya, Kuala Lumpur, Malaysia; AP-HP, Hôpital Raymond Poincaré, Service de Microbiologie et Hygiène, Garches, France; Laboratoire de Biologie intégrative des populations, Evolution moléculaire, Ecole Pratique des Hautes Etudes, Paris, France; Institut de Systématique, Evolution, Biodiversité, ISYEB, UMR 7205, CNRS, MNHN, UPMC, EPHE, Muséum national d’Histoire naturelle, Sorbonne Universités, 16 rue Buffon, F-75231 Paris, Cedex 05 France

## Abstract

**Background:**

In mycobacteria, conjugation differs from the canonical Hfr model, but is still poorly understood. Here, we quantified this evolutionary processe in a natural mycobacterial population, taking advantage of a large clinical strain collection of the emerging pathogen *Mycobacterium abscessus* (MAB).

**Results:**

Multilocus sequence typing confirmed the existence of three *M. abscessus* subspecies, and unravelled extensive allelic exchange between them. Furthermore, an asymmetrical gene flow occurring between these main lineages was detected, resulting in highly admixed strains. Intriguingly, these mosaic strains were significantly associated with cystic fibrosis patients with lung infections or chronic colonization. Genome sequencing of those hybrid strains confirmed that half of their genomic content was remodelled in large genomic blocks, leading to original tri-modal ‘patchwork’ architecture. One of these hybrid strains acquired a locus conferring inducible macrolide resistance, and a large genomic insertion from a slowly growing pathogenic mycobacteria, suggesting an adaptive gene transfer. This atypical genomic architecture of the highly recombinogenic strains is consistent with the distributive conjugal transfer (DCT) observed in *M. smegmatis*. Intriguingly, no known DCT function was found in *M. abscessus* chromosome, however, a p-RAW-like genetic element was detected in one of the highly admixed strains.

**Conclusion:**

Taken together, our results strongly suggest that MAB evolution is sporadically punctuated by dramatic genome wide remodelling events. These findings might have far reaching epidemiological consequences for emerging mycobacterial pathogens survey in the context of increasing numbers of rapidly growing mycobacteria and *M. tuberculosis* co-infections.

**Electronic supplementary material:**

The online version of this article (doi:10.1186/s12864-016-2448-1) contains supplementary material, which is available to authorized users.

## Background

Clonal evolution was a long lasting paradigm in mycobacterial research with the highly clonal flagship of the genus, *Mycobacterium tuberculosis*. Ultimately the rule turned-out to be the exception and true clonal species are rather rare; the most representative members except *M. tuberculosis* are *Yersinia pestis*, *Salmonella typhi* and *Burkholderia mallei*. However, there is increasing evidence that horizontal gene transfer (HGT) and homologous DNA recombination play an important role in the evolution of smooth tubercle bacilli [1, 2] and *M. tuberculosis* strains [3–5]. Gene transfer networks mostly involving genes related to niche change and antibiotic resistance are significantly shaping the adaptive landscape of pathogenic mycobacteria, therefore deciphering the mechanisms behind these empirical observations becomes mandatory. Moreover, knowledge of such mechanisms can shed new light on mycobacterial evolution from saprophytic/commensal organisms to opportunistic or specialized, highly persisting pathogens [3, 6]. However, knowledge about HGT and homologous recombination mechanisms in mycobacteria are scarce. Studies on the saprophytic laboratory model *Mycobacterium smegmatis* showed that chromosomal DNA transfer is mechanistically different from classical Hfr chromosomal DNA transfer, with multiple and wide-spread transfer initiations events from a donor chromosome [7, 8]. This process, called distributive conjugal transfer, creates extensive genome-wide mosaicism within individual transconjugants that generates large-scale sibling diversity conferring the evolutionary benefits of sexual reproduction in an asexual organism [9, 10]. The chromosomal region involved in this unique conjugation mechanism is the ESX-1 secretion system [11], which is also involved in virulence in *M. tuberculosis* [12].

In the present study, our interest focused on *M. abscessus* (MAB). This mycobacteria is an excellent model to study HGT, homologous recombination and their contribution to pathogenicity in mycobacteria. MAB is an emerging opportunistic pathogen, able to cause lung diseases to immunocompetent individuals and that shares a number of characteristics with *M. tuberculosis*, such as the ability to induce granulomatomatous lesions with epithelioid giant cells, caseous necrosis, and silent persistence for decades within host [13]. Since the late 1990’s, MAB has been increasingly recovered from patients with cystic fibrosis (CF) in Europe, Asia and North America [14–16]. Together with *M. avium*, MAB represents the most commonly isolated non-tuberculous mycobacteria (NTM) from CF lung patients. Reports show that MAB isolated from CF patients can account for up to 56 % of all isolated NTM [17]. Compared with other NTM causing pulmonary diseases, MAB can be considered as the most pathogenic, since all reports show that this NTM has the highest rate of genuine ‘clinically relevant’ infections in CF patients according to criteria established by the American Thoracic Society (ATS) [18–22]. Antibiotic resistance is also a major factor in the high rate of treatment failure for MAB pulmonary diseases (20 to 52 %) [23–25]. MAB lung infections cause decline of lung function [17], and dissemination of the infectious agent, eventually leading to death [26, 27]. Moreover, MAB is resistant to nearly all antibiotics, including first-line antitubercular drugs [28], and the few active antibiotics only seem to have a bacteriostatic effect [29]. Taken together, these features make MAB an emerging pathogen under close surveillance. Furthermore, MAB is also satisfying population studies criteria, since large isolate collections are available, with systematically documented clinical profiles and cohort studies, especially in the case of cystic fibrosis (CF) patients [18].

Another characteristic making MAB a very relevant model for genetic exchange study in mycobacteria is the fact that it harbors various phenotypes such as rough and smooth morphotypes [30], macrolid resistance [31], and anaerobic growth [32]. MAB encompasses a large genetic diversity [33] that is markedly associated with different prevalence [34–36], specific involvement in outbreaks [37], distinct virulence [38] and contrasting clinical outcomes [39].

Recent genomic studies comparing the genomes of 40 strains from the MAB unravelled a large repertoire of accessory genes, suggesting extensive genetic acquisition capacities and high evolutionary potential for this species by HGT [40]. This trend was confirmed with the publication of a reference genome sequence obtained by Sanger method, showing that some virulence genes might have been acquired by HGT from non-mycobacterial species sharing a similar ecological niche [41]. Moreover, MAB is subdivided into at least two recognized subspecies: *Mycobacterium abscessus subsp. abscessus* and *M. abscessus subsp. bolletii* [42, 43], making this species an excellent model for studying intra-specific homologous recombination. Multi Locus Sequence typing (MLST) studies showed that some clinical isolates have a composite genetic pattern with housekeeping genes corresponding to different subspecies, suggesting that homologous recombination occurs readily within the MAB [34, 35, 44, 45]. However, unlike *M. smegmatis*, sequence analysis of 14 MAB genomes showed that no ESX-1 orthologous system is encoded within this species, whereas ESX-3 and ESX-4 secretion systems are present [46], raising the question whether alternative DCT systems might exist. Studying mycobacteria-specific HGT dynamics and the involved cellular machinery might definitively improve our understanding of the subspecies border delimitations and the amount of gene flow occurring within the MAB.

Although HGT (exogenous insertions as well as inter-strain homologous recombination) seem to be extensive in MAB strains, no quantitative data are available regarding the genetic flux between specific MAB subspecies, and the genetic architecture (i.e., location, distribution, and extent) of these genetic exchanges on the genome are unknown. Thus, using an MLST approach, and taking advantage of a set of 280 clinical strains, our goal was to extensively characterize the genetic exchanges occurring between MAB subspecies. This approach led us to identify a sub-population of highly admixed strains. Furthermore, using comparative genomics, we succeeded in generating comprehensive recombination cartography for some of these admixed strains. This highly admixed sub-population was then further investigated for virulence by using clinical records. Finally, in-depth genomic analysis was performed in order to identify putative specific DCT functions.

## Results

### Phylogenetic signal and analyses

Prior conducting any phylogenetic inferences based on the 7 MLST gene fragments (*argH*, *Cya*, *glpK*, *gnd*, *murC*, *pta*, and *purH*), we inferred the quality of the phylogenetic information contained in these sequences by plotting transition and transversion rates as a function of genetic distances (Additional file 1: Figure S1). This graph shows that neither transitions nor transversions are saturated but, rather, both rise linearly with increasing genetic distance. We then measured substitution saturation using the Xia index [47] for all three codon positions. The observed *I*_*ss.c*_ value of 0.808 was significantly higher than the *I*_*ss*_ value of 0.019, thus confirming that little saturation occurs at these sites.

**Fig. 1:**
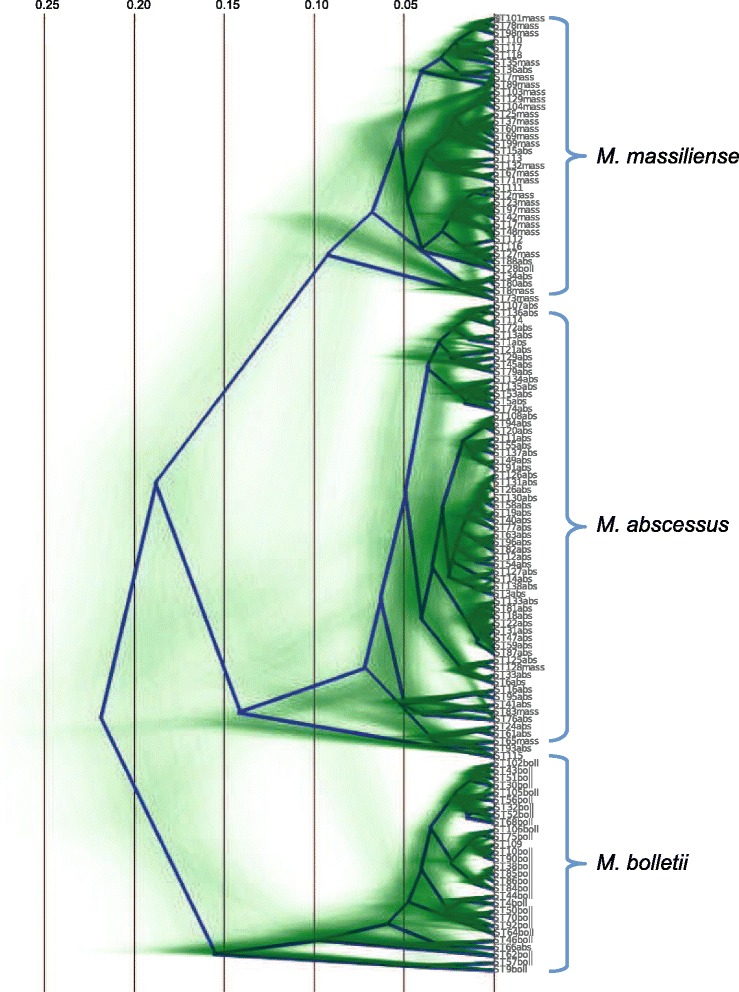
Densitree of 130 STs belonging to the MAB. The phylogenetic reconstruction is based on the concatenated housekeeping genes (3576 bp) and the GTR + I + G evolutionary model was implemented in BEAST 1.7.5. The best 8000 trees generated during the Markov chain are drawn transparently (*in green*). As a result, areas where the vast majority of the trees agree in topology and branch lengths show up as deep green, while areas with little agreement show up as webs. The root canal tree (*blue lines*) has a topology that is selected from the set of trees and has the highest probability of clades in the set. The scale corresponds to the genetic distance

The evaluation of the phylogenetic content of the dataset was performed with likelihood mapping analyses. The results point to a robust phylogenetic signal (>96 %), however a low amount of star-likeness with ~1 % of all quartet points in the central region of the triangle indicates that certain parts of the tree are unresolved (Additional file 2: Figure S2). These unresolved nodes could be visualized using a densitree, which plots 1000 different topologies retained during the Monte Carlo Markov Chain in BEAST (Fig. 1). Though a real support exists for the three main lineages, many alternative topologies were retained, illustrating information conflicts and therefore suggestive of HGT.

### Population genetics and recombination

**Fig. 2:**
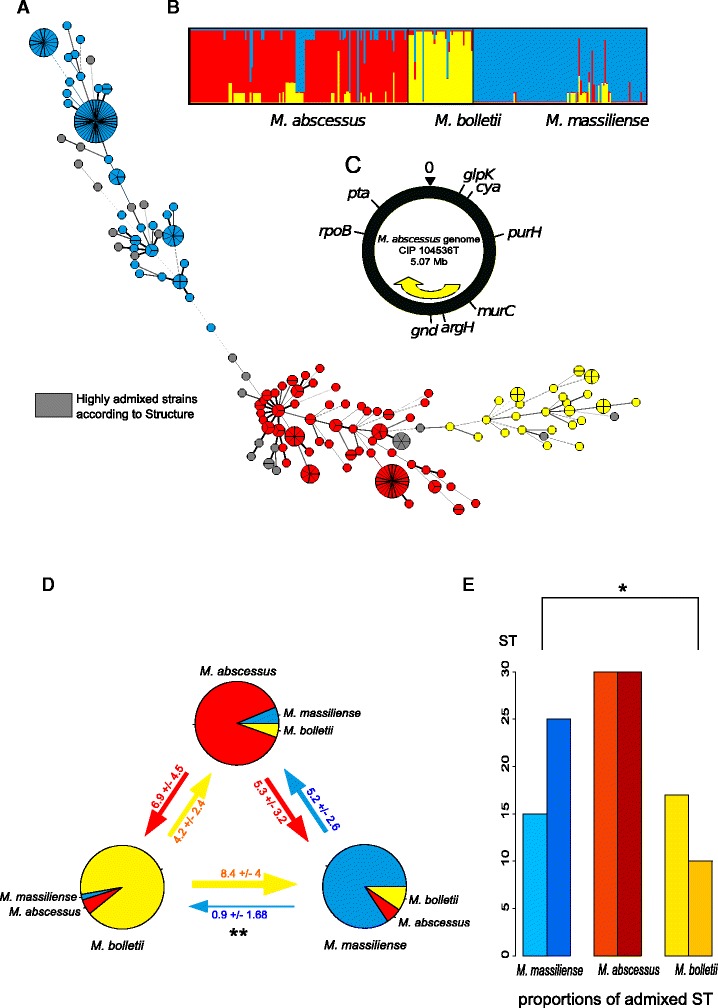
Ancestry of 280 strains belonging to the MAB. **a** Distribution of the three subspecies (plus admixed strains) within a minimal spanning tree (MS_TREE_) based on the degree of allele sharing. Circles are proportional to the number of strains and are colored according to the subspecies identification obtained from the Bayesian algorithm STRUCTURE. The thickness of the line is function of the number of shared alleles. **b** Proportion of ancestry from the subspecies *M. abscessus*, *M. bolletii* and *M. massiliense* as inferred by STRUCTURE and their assignment to three *rpoB* groups as displayed with DISTRUCT [78]. **c** Genomic locations of the 7 housekeeping genes and the diagnostic genes *rpoB*. **d** Synthetic network representing the amount of gene flow between the three subspecies based on the sole STs and the STRUCTURE assignments. For each subspecies, percentage of foreign allele (form other subspecies) is indicated with 99 % confidence interval. **e** Proportions of admixed ST for each subspecies. Left bar: not admixed (*ie*. no exogenous allele). Right bar: at least one exogenous allele (*ie*. from another MAB subspecies). *P < 0.05; **P < 0.01 (Fisher exact two-sided test)

Neighbor-nets were inferred in order to detect putative recombination signatures that will result in networks. Neighbor-nets calculate networks of multiple alternative pathways between taxa whenever homoplasy or recombinations result in phylogenetic inconsistencies. The analysis of the concatenated genes recovered the same phylogenetic clusters as the traditional phylogenetic approaches (Additional file 3: Figure S3). However, reticulations are extremely common between all three species, suggesting that recombination is a major driving force in the MAB. This observation was independently confirmed by a significant PHI test [48] (*P* < 0.001). Therefore we decided to implement a population genetic approach by analysing the polymorphisms present in all seven-gene fragments (Fig. 2c) with STRUCTURE, which employs a Bayesian method to discern groupings among recombining organisms. The linkage model of STRUCTURE assigns probabilities of derivation from ancestral source groups for each polymorphic nucleotide. The ancestry of each strain is then estimated as the summed probability of derivation from each group over all polymorphic nucleotides. STRUCTURE recognized three ancestral sources of polymorphisms within the MAB (K = 3 populations shows a clear inflection in the likelihood values) and separated most *M. abscessus*, *M. bolletii* and *M. massiliense* isolates into those groups (Fig. 2b). Those results highlight that the separation into three groups is the most valuable, and thus we will subsequently use the terms *M. abscessus*, *M. bolletii* and *M. massiliense* to identify those 3 different groups of MAB strains in the following text sections. However, within the entire dataset, numerous strains contained significant ancestry from multiple sources, highlighting therefore their mixed ancestries. We assigned ~90 % of the strains, whose proportion of nucleotides from one of the three ancestral sources exceeded a threshold value of 80 % to the subspecies *M. abscessus*, *M. bolletii* and *M. massiliense* and one tenth (29/280) to hybrid profiles (Fig. 2a).

### Allelic flux

After having attributed each sequence type (ST) to a given subspecies, we determined the percentage of exogenous SNPs for each ST (Fig. 2d). Subspecies belonging to *M. massiliense* and *M. abscessus* strains display the highest proportion of foreign SNPs, whereas *M. bolletii* strains are far more homogenous. Moreover, allelic flux going from *M. massiliense* to *M. bolletii* was significantly lower than other allelic exchanges (Fisher test *P* < 0.01. Fig. 2d). Consistently, mosaic strains proportion is significantly higher for *M. massiliense* STs than for *M. bolletii* (Fisher test *P* < 0.05. Fig. 2e). Although they should be confirmed by whole genomic survey, these results suggest that allelic fluxes between the three subspecies are not homogenous. They indicate asymmetrical gene flow between subspecies, especially between *M. massiliense* and *M. bollettii*.

We also sought to quantify the frequency of homologous recombination within groups by implementing the composite likelihood of *r*/*μ* (the probability that a nucleotide will change by recombination divided by the probability that the same nucleotide will change by mutation) [49]. The test confirmed that significant levels of recombination had occurred within each subspecies (Table 1). Interestingly, if we consider results from the concatenated housekeeping genes (that allow to integrate large-scale inter-locus recombination), recombination estimates raise dramatically for *M. abscessus* and *M. massiliense*. Taken together, these data suggest that the main driving evolutionary force is recombination and not mutation in *M. abscessus* and *M. massiliense*, and that these recombination events occur at genomic scale involving large inter-locus fragments.

**Table 1:** Population estimates of mutation rates (*θ*) and recombination rates (*ρ*) per base

	*θ*				*ρ*				*r / μ*			
	*M. abs*	*M. bol*	*M. mas*	MAB	*M. abs*	*M. bol*	*M. mas*	MAB	*M. abs*	*M. bol*	*M. mas*	MAB
*argH*	0.007	0.006	0.015	0.018	0.005	0	0	0.001	0.714	0	0	0.056
*cya*	0.001	0.012	0.011	0.012	0.004	0.016^a^	0.004	0.008^a^	4	1.333^a^	0.364	0.667^a^
*glpK*	0.007	0.008	0.006	0.01	0.002	0.011	0.001	0.004	0.286	1.375	0.167	0.4
*gnd*	0.004	0.012	0.014	0.016	0.005	0	0.006	0.004^a^	1.25	0	0.429	0.25^a^
*murC*	0.009	0.012	0.008	0.015	0	0	0.001	0	0	0	0.125	0
*pta*	0.009	0.006	0.008	0.01	0.016^a^	0	0.012^a^	0.006	1.778	0	1.5	0.6
*purH*	0.011	0.003	0.011	0.012	0.007^a^	0.008^a^	0.005^a^	0.005	0.636	2.667	0.455	0.417^a^
Average.	0.007	0.008	0.01	0.013	0.006	0.005	0.004	0.004	0.813	0.593	0.397	0.29
Concat^.b^	0.008	0.008	0.011	0.013	0.041^c^	0.009	0.03 ^c^	0.004	5.125^a^	1.125 ^a^	2.727^a^	0.308^a^
CI 95 %					0.038–0.044	0.008–0.010	0.028–0.032	0.004–0.004	4.75–5.5	1–1.25	2.55–2.9	0.308–0.308

The analyses are realised within lineages and for the MAB complex (pooled genotypes)

Values for rho (*ρ)* were obtained by dividing the per-locus recombination rate estimate from LDhat by the sequence length. *μ:* mutation rate per nucleotide; *r*: recombination rate per nucleotide*.* ^a^: significant recombination estimates. Concat. ^b^concatenated data set. ^c^: recombination significantly higher than intra-genic recombination rates (^a^ and ^c^: 95 % confidence interval)

### rpoB typing

A side effect of the allelic flux within MAB concerns the suitability of *rpoB* for subspecies identification. Indeed, there is no reason that this gene will escape interlineage homologous recombination and consequently using it as a diagnostic marker for species determination within the MAB might be misleading (Additional file 4: Figure S4A). According to STRUCTURE assignments, and consistent with our previous result emphasizing *M. massiliense* high recombination rates, the false group identification rate is about 10 % within MAB and climbs up to 20 % for strains belonging to *M. massiliense* (Additional file 4: Figure S4B).

### Clinical symptoms of infection and mosaicism

**Fig. 3:**
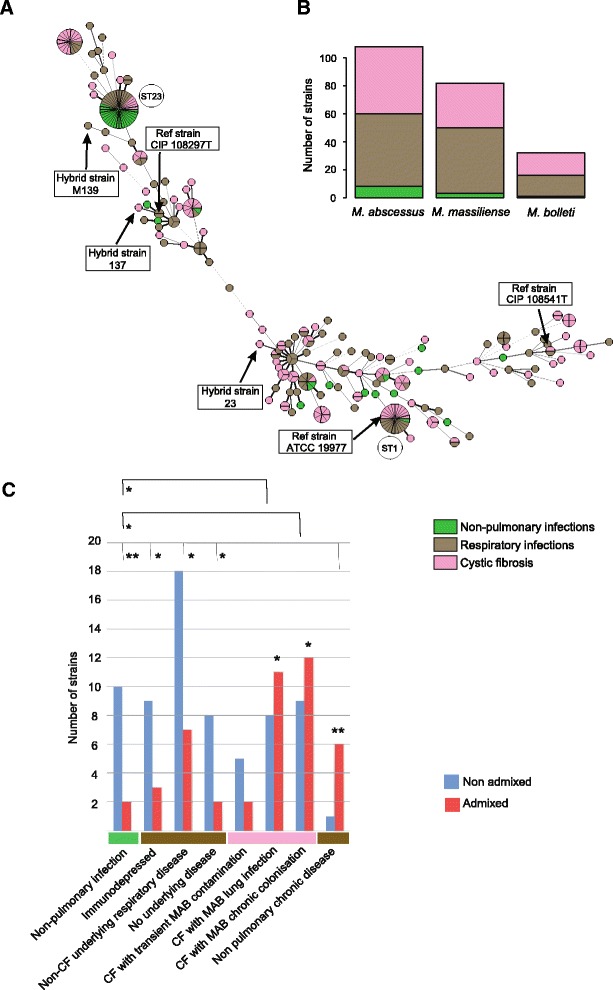
Pathogenic types within an MS_TREE_ and distribution of admixed strains according to clinical profiles. **a** Each strain is represented by a circle or a fraction of a circle, and colours correspond to different pathogenic types. Circled numbers indicate frequently encountered ST complexes or outbreak strains. Black lines connecting pairs of STs indicate that they share six (thick lines), five (thin) or four alleles (dotted) out of seven. Boxed strains correspond to the three MAB reference strains. Moreover, they include three additional admixed strains selected for whole-genome sequencing and comparative analyses. **b** Pathogenic type distribution according to the three subspecies (the frequencies did not differ significantly). **c** Admixture spectrum frequency according to the principal clinical profiles. Non-admixed: strain with absence of exogenous allele. Admixed: strain with at least one exogenous allele coming from another subspecies. *P < 0.05; **P < 0.01 (Fisher exact two-sided test)

Allele-based relationships within the MS_TREE_ also allowed us to address whether virulent phenotypes are lineage specific. None of the clinical profiles (non-pulmonary infections, respiratory infections and cystic fibrosis) is restricted to one of the three subspecies (Fig. 3a). Moreover, statistical analyses did not detect any geographical or phenotypic association with one of the members of the MAB.

Clinical information was collected from 102 different MAB isolates from infected patients with well-documented geographic origins and clinical background (See Additional file 5: Table S1). The patients were divided into sub-groups according to the type of infection (non-pulmonary *vs* pulmonary infections), and within pulmonary infections patients were divided according to the underlying clinical context (immunocompromised patient, respiratory underlying disease, non-respiratory chronic disease, and cystic fibrosis). Within this sample, CF patients were gathered from a former multi-center study, and were monitored on several years (1996–2009), allowing the identification of chronic MAB colonisation. These patients were also precisely identified according to the clinical manifestation of non-tubercular lung disease (NTMLD) showing clinical symptoms suggestive of lung infection. An exogenous allele acquisition index (inter subspecies) was computed for each isolate, using STRUCTURE and *rpoB* results to determine if at least one of the eight sequenced genes came from exogenous subspecies. The results show that strains belonging to mosaic STs are over-represented in CF patients with MAB infection or chronic lung colonisation (Fig. 3c). Whereas CF patients with MAB infection or chronic colonisation had a majority of admixed MAB isolates, most non-CF patients were infected with low proportion of admixed MAB strains (except in the case of patients with non-pulmonary chronic disease). These proportions differ significantly (Fisher exact test *P* = 0.038 for CF patients with MAB chronic colonisation, and *P* = 0.034 for CF patients with MAB lung infection). More specifically, the proportion of admixed MAB significantly differed between patients with non-respiratory infections, as compared with CF patients with MAB infection or chronic colonisation (Fisher exact test *P* = 0.03 for both patient category). The most marked difference was observed for patients having MAB pulmonary infection with non-pulmonary underlying chronic disease. MAB associated with those patients very significantly differed from other stains (Fisher exact test *P* = 0.008). In order to exclude potential cross-contamination effect, the same calculations were performed with only single STs, and the differences observed between the most extreme distribution was still significant (for patients having pulmonary infection with non-pulmonary underlying chronic disease : Fisher exact test *P* = 0.029 as compared to patients with Non-pulmonary infections, and *P* = 0.039 as compared to all other patients). Those results suggest that different MAB populations, showing contrasted patterns of genetic admixture, are preferentially associated with some specific clinical profiles (depending on infection types and patient clinical background).

### HGT mapping and comparative genomics

**Fig. 4:**
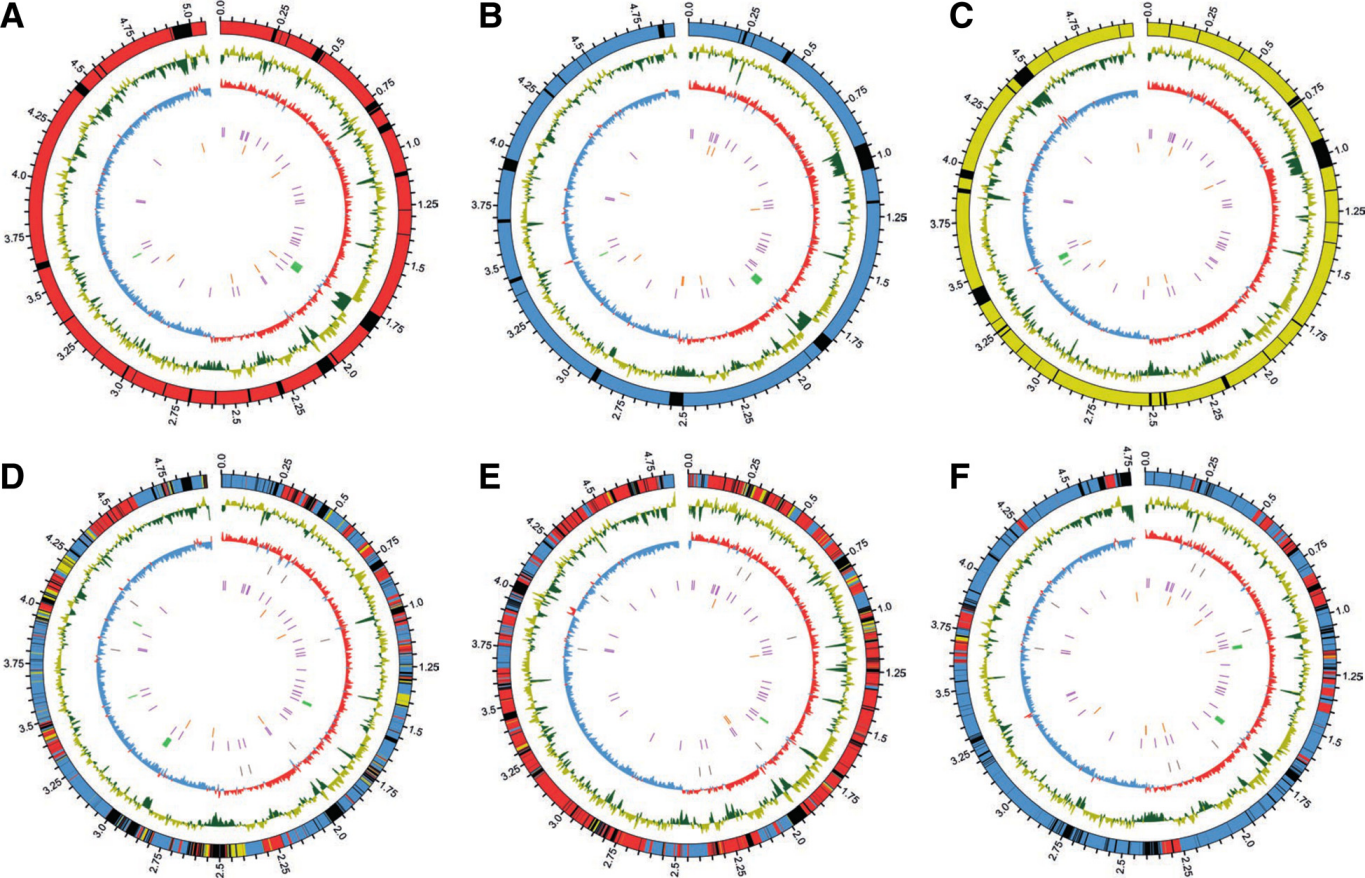
Circular representation of MAB subspecies reference and admixed strains. **a**: *M. abscessus*, **b**: *M. massiliense*. **c**: *M. bolletii*. **d**: *M. massiliense* strain M139, **e**: *M. abscessus* strain 137, **f**: *M. massiliense* strain 23. For the outer circle, colours were reported following subspecies identification (red: *M. abscessus*, blue: *M. massiliense.* yellow: *M. bolletii*, black: regions with no significant subspecies attribution or not belonging to core genome). From outer to inner circle: subspecies type identification; GC% in 5-kb non-overlapping windows (yellow: higher than 64 %; green: lower than 64 %); GC skew in 5-kb non-overlapping windows (red: positive; blue: negative); sequenced genes for identification studies (MLST genes and *rpoB*) in grey; prophage regions in green; tRNA in magenta; and IS element in orange. Circular plots were generated by using CIRCOS [79]

To obtain a genome-wide perspective of the processes detected using the MLST approach, we decided to fully sequence three genomes (M139, 23 and 137, see Fig. 4) that might be massively affected by inter-strain homologous recombination and HGT and to compare their genomic architecture with three reference genomes (Additional file 6: Figure S5 and Table 2). Based on the reference strains, species-specific SNP density plots were constructed (Additional file 7: Figure S6) and we were able to attribute subspecies identity to most of the admixed strain genome positions. The assembled genomes of strains M139, 23 and 137 displayed sequence identity with the corresponding reference genomes over 80, 83 and 78 % of the sequence, respectively, suggesting that a large proportion of the core genome was recovered in genomic alignment of these strains (Fig. 4). These strains also had respectively 15, 11 and 13 % of their genome belonging to the accessory genome (genomic islands and strain specific genes). Strain M139 showed a large overlap with *M. massiliense* (63 % of the genome), whereas 18 % of the sequence was identical to *M. abscessus* and 8 % to *M. bolletii* (Fig. 4d). Strain 137 was in majority identical to *M. abscessus* (54 % of the genome) with another 21 and 3 % identical to *M. massiliense* and *M. bolletii,* respectively (Fig. 4e). Finally, Strain 23 was mainly identical to *M. massiliense* (70 % of the assembled genome) and 13 % identical to *M. abscessus*. This strain presented nearly no trace of *M. bolletii-*like sequences (Fig. 4f). These comparative genomic results are congruent with our previous results gathered from the Bayesian STRUCTURE program. Furthermore, the peripheral position of the selected strains on the MS_TREE_ is clearly the product of genetic admixture.

**Table 2:** Core, variable, and strain specific open reading frames (ORFs)

Organism	ORFs	Core ORFs	Var ORFs	Strain spe. ORFs	Core ORFs (%)	Var ORFs (%)	Strain spe. ORFs (%)
*Mycobacterium abscessus* ATCC 19977	5477	4160	1317	617	76	24	11.3
*Mycobacterium massiliense* CIP 108297 T	5247	4135	1112	249	78.8	21.2	4.7
*Mycobacterium bolletii* CIP 108541 T	5275	4153	1122	520	78.7	21.3	9.9
*Mycobacterium massiliense* strain M139	5269	4145	1124	435	78.7	21.3	8.3
*Mycobacterium abscessus* strain 137	5267	4136	1131	459	78.5	21.5	8.7
*Mycobacterium massiliense* strain 23	5332	4122	1210	481	77.3	22.7	9

ORFs were identified on MAGE plateform, and core genome was defined by Bidirectional Best Blast Hit (BBH) with a 50 % sequence identity threshold, and at least 80 % coverage

Large recombination domains (up to 93 kb) are also found all along the genomes, and may account for up to a quarter of the genomic content. Interestingly, chromosomal mapping domains that are identical to each of the reference strains show a non-random distribution. These domains are clustered together into large continuous regions that are associated with one of the three reference strains (Fig. 4 and Additional file 7: Figure S6). For example, in strain M139, the length of the largest domain similar to *M. abscessus* was 53 kb (median length 5.4 kb); the length of the largest domain similar to *M. bolletii* was 48 kb (median length 4 kb) and the length of the longest domain similar to none of the reference strains was 61.5 kb (median length 4 kb). Worth mentioning, our results confirm that strain M139 belongs to the *M. massiliense* subspecies and harbours an *M. abscessus* subspecies *erm* (41) allele (usually conferring inducible macrolide resistance) [50, 51]. This *erm*(41) element is embedded in a large continuous 60 kb genomic region also clearly assigned to an *M. abscessus subspecies* genetic background (Additional file 8: Figure S7A). Interestingly, the same M139 highly mosaic strain also contained a large non-aligned contig containing a locus highly similar to the *M. marinum* p-RAW conjugative mega-plasmid (Additional file 8: Figure S7B) [39, 52]. Thus, our results show that MAB population is structured into three sub-species, and also contains some unclassifiable mosaic strains. These unclassifiable individuals are highly plastic (that have almost half of their genomic content remodelled by HGT) with a pattern that strikingly resembles DCT genome mosaicism reported in *M. smegmatis* and *M. canettii* [2].

## Discussion

Here, we sampled and analysed a representative and balanced collection of strains that represents the MAB diversity, both at phylogenetic and epidemiological levels, and from different geographical sources. Our results obtained from MLST analyses combined with whole genome sequence analysis of some representative strains clearly confirm the existence of 3 subspecies and therefore cast some doubts on the classification proposed by Leao and colleagues [53]. These authors suggested to group *M. bollettii* and *M. massiliense* together into a subspecies named *M. abscessus subspecies bolletii* comb. nov.

On the other hand, according to the large amounts of putative gene flow detected by STRUCTURE and LDHAT (Table 1), the three entities described here do not fully fit the genospecies described by Drancourt and colleagues [46]. Indeed, our MLST data suggest that homologous recombination in the MAB has been frequent enough to generate about one third of strains within the species with traces of mixed ancestries (Fig. 2b), from which another third (so-called admixed strains) have at least 20 % of their gene content from a foreign source. Even more striking is the fact that significant homologous recombination was detected both within and between subspecies (Table 1). This means that the MAB does not fit into a clonal framework and that the amount of genetic exchange detected here resembles the one reported in a previous study on *Escherichia coli* [54]. However, the data show that the three subspecies do not behave the same way. Indeed, *M. bolletii* is clearly less introgressed than the two other subspecies, resulting in a relatively homogenous gene pool that might result from a distinct or isolated ecological niche. It is tempting to link this observation with the rather low prevalence of *M. bolletii* in cystic fibrosis [18] and other chronic pulmonary infections [19]. Moreover, the limited genic repertoire of this subspecies combined with its mostly clonal propagation will limit its adaptive landscape in a clinical setting.

The situation is dramatically different for *M. abscessus* and *M. massiliense*, where homologous recombination is prevalent. For example, in *M. abscessus*, each nucleotidic change has nearly seven more chances to be generated by recombination than by mutation. There is accumulating evidence that recombinogenic species display higher virulence profiles and plasticity than clonal ones [54, 55]. This situation is encountered in the MAB, where isolates gathered from CF patient with record of clinical symptoms have exchanged significantly more alleles between subspecies than isolates from patients with other clinical profiles (Fig. 3c). This observation might also be linked to the fact that the majority of MAB infections in CF patients are silent, whereas only 10 % of the isolates are associated with clinical symptoms of pulmonary disease (Roux et al. in press), which is reminiscent of the 10 % of hyper-mosaic strain observed. Taken together, these observations show a link between chronic lung colonization and mosaicism in MAB. This could be driven by different scenarios: MAB mosaic strains might have acquired genes or alleles conferring greater virulence profile and/or lung colonization ability, alternatively the specific conditions associated with pulmonary tissue colonisation (such as host defence, or drug treatments) may submit colonizing strain to environmental stresses that favour genetic exchanges.

From a clinical point of view, our study does not provide any trend or information that favours one type of subspecies in a given clinical situation. Furthermore, soft tissue infections, lung diseases and systemic diseases were not preferentially associated with any subspecies. This absence of clinical and geographical correlations confirms the opportunistic, accidental nature of the infections, most likely from an environmental source. In terms of clinical diagnostics, we clearly illustrate the lack of power of *rpoB* typing that culminates at 20 % failure rates in the *M. massiliense* subspecies. Accordingly, molecular diagnostics might definitely profit from a multilocus typing scheme.

MLST has proven to be a powerful screening tool in molecular epidemiology [56]. However, the use of next-generation sequencing (NGS) for full-genome sequence determination by far extends the amount of information gathered by MLST. Therefore we decided to initiate a comparative genomic approach that combines three reference strains from each subspecies with three genomes identified as admixed in the Bayesian analyses. A Venn diagram shows that within the two most recombinogenic lineages, the *M. massiliense* reference strain has the smallest repertoire of genes within accessory genome (Additional file 6: Figure S5), a feature that might correlate with its milder pathogenic profile [36, 39]. Correlatively, in admixed strain genomes, numerous regions of up to 40–60 kb in size, containing many non-core genes and displaying GC% variation (Fig. 4 and Table 3) were detected, providing excellent candidates for putative inserted genomic islands. This is the case of strain M139 harbouring a clear *M. intracellulare*-like region (Table 3). These regions are distributed all along the genome, and represent 11 to 15 % of the circular chromosome. However concomitant acquisition of such genomic islands with the DCT-like genomic exchanges observed remain to be investigated.

**Table 3:** Nucleotide BLAST results of admixed genomes genomic islands

Strain Query	Genomic island Start position	Genomic island End position	Length (bp)	First nucleotide blast result^a^	Total score	Query cover	E.value	Max
*Mycobacterium massiliense* strain M139	1922873	1992300	69427	*Mycobacterium intracellulare* MOTT-02, complete genome^b^	1.12E + 005	99 %	0	99 %
*Mycobacterium massiliense* strain M139	4564885	4576047	11162	*Mycobacterium sp*. JDM601, complete genome ^b^	8504	74 %	0	80 %
*Mycobacterium massiliense* strain M139	2800023	2810210	10187	*Mycobacterium smegmatis* JS623, complete genome	3918	77 %	0	81 %
*Mycobacterium massiliense* strain M139	254418	263728	9310	*Mycobacterium chubuense* NBB4, complete genome	1960	38 %	0	84 %
*Mycobacterium massiliense* strain 23	870688	892899	22212	*Mycobacterium avium* 104, complete genome	21406	63 %	0	99 %
*Mycobacterium massiliense* strain 23	2385439	2401289	15851	*Mycobacterium gilvum Spyr1, complete genome*	10443	56 %	0	89 %
*Mycobacterium abscessus* strain 137	1922873	1992300	69428	*Mycobacterium intracellulare* MOTT-02, complete genome^b^	1.07E + 005	88 %	0	99 %
*Mycobacterium abscessus* strain 137	2172909	2194917	22009	*Mycobacterium sp*. JLS, complete genome	3410	41 %	0	98 %
*Mycobacterium abscessus* strain 137	4564885	4576047	11163	*Mycobacterium ulcerans* Agy99, complete genome^b^	2879	55 %	0	93 %

^a^First nucleotide blast result with more than 80 % identity, and covering at least 30 % of the sequence of the tested Genomic Island

^b^pathogenic mycobacteria

The genetic architecture of the admixed strains advocates for rather rare but massive genetic exchanges between MAB subspecies and only hardly fits with long-lasting and regular gene flow that would lead to highly scattered patterns disrupting the genome-wide mosaicism into a gene-wide mosaicism. In the context of an absence of ESX1 loci in our MAB collection and with no evidence that ESX3 or ESX4 play a role in *M. smegmatis* DCT (which also contains those two types of ESX), the genetic elements involved in MAB mosaicism remain unresolved. However, the two extrachromosomal elements carrying ESX/typeIV systems detected in some MAB strains share many traits with the novel *p-RAW* plasmids discovered in *M. marinum* and other SGM [52, 57] (Additional file 8: Figure S7B). Recent investigations also showed that other similar ESX/typeIV systems exist in mycobacteria and belong to a quite large and diversified family. Taken together, these results raise the hypothesis that, besides the genomic ESX1-driven DCT, other extrachromosomal p-RAW-like ESX elements might be involved in mycobacterial conjugation, and allow subsequent DCT. We are aware that this novel hypothetical form of plasmid-driven conjugation must be tested in experimental F1-generation transconjugant experiments to evaluate the evolutionary and pathogenic potential behind this system in MAB.

## Conclusion

Admixed populations of MAB seem to display higher abilities for colonizing lungs of CF patients. On the other hand, we cannot exclude that long-term lung colonization might also favour MAB genetic admixture and HGT in this very specific ecological niche. Our study also strongly suggests that pRAW-like extra-chromosomal genetic elements might be responsible for the massive genomic exchanges observed in MAB, and are reminiscent of those observed in distributive conjugal transfer coded on chromosomal ESX1 system in *M. smegmatis*. Therefore, their contribution to HGT in mycobacterial evolution and pathogenicity should be assessed in a general context of increasing MAB lung infections and MAB/TB co-infections.

## Methods

### Bacterial strains collection and sequence dataset

A total of 280 strains belonging to the MAB were chosen from multiple clinical sources in diverse geographical areas in an attempt to study the genetic diversity of this bacterial species from an evolutionary perspective (Additional file 5: Table S1). In order to verify that all strains belonged to MAB, *RpoB* typing was performed, and phylogenetic trees of each of the seven housekeeping genes used for MLST analysis (*argH*, *Cya*, *glpK*, *gnd*, *murC*, *pta*, and *purH*), were carefully checked (see Additional file 9: Figure S8). We also assessed that the associated housekeeping genes were under strong purifying selection (Additional file 10: Figure S9). To estimate the level of gene conservation, pairwise dN/dS ratio ω (dN: non-synonymous mutation substitution rate, dS: synonymous mutation substitution rate) were calculated using the program CODEML provided by the PAML (Phylogenetic Analyses by Maximum Likelihood) package version 4 [58]. Nucleotidic sequences have been aligned using TRANSLATORX [59] guided by protein sequence alignments obtained using M-COFFEE [60].

### Clinical information

Clinical information was collected from 102 different MAB isolates from different infected patients with precise documented geographic origins and clinical background (See Additional file 5: Table S1). In order to avoid samples biases, we only used one isolate per patient and per reported outbreak. These patients were divided into sub-groups according to the type of infected tissue (non-pulmonary *vs* pulmonary infections). Within pulmonary infections patients were divided according to the underlying clinical context (immunocompromised patient, respiratory underlying disease, non-respiratory chronic disease, and cystic fibrosis). CF patients clinical profile were precisely documented from a French multicenter cohort study [18]. All the patients, or their parents if they were children, gave their informed consent. Data were retrieved from the French CF registry (CNIL authorisation No 1,202,233), and an internal review board approved the study. Samples were analyzed for NTM identification at each center, using approved techniques and all data analysed were anonymized. CF patients were considered as infected if MAB was associated with “nontuberculous mycobacterial lung disease” (NTMLD), if the cases: i) fulfilled the bacteriological American Thoracic Society criteria for mycobacterial lung infections [61], and ii) presented clinical (e.g., functional deterioration such as fever, asthenia and emaciation) and/or radiographic signs of mycobacterial disease. Each CF patient was periodically investigated for pulmonary MAB isolation between 1996 and 2009. If MAB was no more identified after previous positive tests, and without any anti-MAB treatment, this was considered as a ‘transient colonisation’. If MAB was isolated at each investigation after first isolation on a minimal period of 5 years, this was considered as a ‘chronic colonisation’.

### Gene fragments sequencing

Eight gene fragments were amplified and sequenced from all isolates using the primers and PCR protocols presented in Macheras et al. 2011. Both strands were sequenced using an Applied Biosystems Prism 3700 automated sequencer with dRhodamine-labeled terminators (PE Applied Biosystems). Sequences were aligned and trimmed using SEQLAB and PILEUP (Wisconsin Package 9.1, GCG, Madison, WI) and then concatenated. All sequence type profiles and nucleotide sequences are publicly available at (http://bigsdb.web.pasteur.fr/mycoabscessus/mycoabscessus.html).

### MLST and minimum spanning-trees

In order to define the relationships between strains at the microevolution level, we performed allelic profile-based comparisons using a minimal spanning tree (MST) analysis with the BIONUMERICS v5.10 software (Applied-Maths, Sint Maartens-Latem, Belgium). The minimal spanning tree is calculated by Prim’s algorithm, modified to choose between otherwise equivalent, alternative subtrees at each step by implementing priority rules that incorporate aspects of the EBURST algorithm [62]. The highest priority is given to STs with the largest numbers of single locus variants. Any ties were resolved by choosing the ST (or a random ST) with the largest number of isolates. The first node in the network is the ST with the highest priority according to these rules and subsequent links are chosen by a recursive strategy. ST complexes were defined as containing at least three STs, with links of one or two shared alleles. The graphical representation displays the quantitative relationships between STs and ST complexes, measured as the number of shared alleles, by lines of different thickness and type.

### Phylogenetic analyses

In order to gain an overview of the phylogenetic signal, we plotted pairwise transition and transversion distances against the total genetic distances using the DAMBE software package [63], and we also tested our set of molecular sequences for substitution saturation. The phylogenetic signal of the dataset was also investigated with the likelihood mapping method implemented in TREE-PUZZLE [64] by analysing 10,000 random quartets. This method proceeds by evaluating, using maximum likelihood, groups of four randomly chosen sequences (quartets). The three possible unrooted tree topologies, for each quartet, are weighted and the posterior weights are then plotted using triangular coordinates, such that each corner represents a fully resolved tree topology. Therefore the resulting distribution of the points shows whether the data are suitable for a phylogenetic reconstruction, or not. The best-fit model of DNA substitution and the parameter estimates used for tree reconstruction were chosen by performing hierarchical likelihood ratio tests implemented in JMODELTEST 2.1.3 [65]. Phylogenetic trees were estimated for each data set with PHYML incorporating the best-fit model of evolution. Alternatively, we also implement the Bayesian Markov chain Monte Carlo (MCMC) method available in the BEAST 1.8.1 package [17] to generate a phylogeny of MAB. The general time reversible (GTR) substitution model was implemented under a constant population size scenario and five independent runs were generated. Convergence was then evaluated with ESS values and trace plots were explored with the software TRACER 1.5. Runs were combined using LOGCOMBINER and trees were plotted using FIGTREE v1.3.2 and DENSITREE 2.0.1. Split decomposition analyses were performed with SPLITSTREE, version 4 [66], by using LogDet distances, equal edge lengths, and 1000 bootstrap replicates.

### Population genetic analyses

We used the linkage model in STRUCTURE [67] to identify groups with distinct allele frequencies [54, 68, 69]. This procedure assigns a probability of ancestry for each polymorphic nucleotide for a given number of groups, *K*, and also estimates q, the combined probability of ancestry from each of the *K* groups for each individual isolate. As given by the Evanno’s test [70], we chose three groups for this report because repeated analyses (200,000 iterations following a burn-in period of 80,000 iterations) with *K* between 1 and 10 showed that the model probability increased dramatically between *K* = 2 and *K* = 3 and only slowly thereafter. A cut-off value of q ≥ 0.80 was then used to assign individual isolates to one of the three groups that largely matched the classical nomenclature. Unassigned isolates were designated as “hybrid” strains.

### Recombination and mutation

We tested for recombination within the 3 subspecies for all loci independently, as well as for the concatenated MLST loci using the software LDHAT v2.2 [49]. LDHAT employs a coalescent-based method to estimate the population-scaled mutation (*θ* = 2*N*_e_*μ*) and recombination (*ρ* = 2*N*_*e*_*r*) rates, where *N*_*e*_ is the effective population size, *r* the rate at which recombination events separate adjacent nucleotides and *μ* is the mutation rate per nucleotide. The ratio *r*/*μ* were calculated as (*ρ*/L)/*θ*, where L is the gene length (sequence length). This *r*/*μ* ratio ranges from 0, which indicates full clonal reproduction, to > > 1, which is expected under free recombination. Significance of the evidence for recombination was tested using non-parametric, permutation-based tests implemented in LDHAT (Lkmax and G4 tests). To avoid strains overrepresentation, the analysis was conducted on the sole STs. Concatenated sequences recombination rates confidence intervals were calculated using likelihood curve method.

### *M. bolletii* and *M. massiliense* reference strains DNA sequencing and genome assembly

The genomic DNAs of *Mycobacterium abscessus subsp bolletii* reference strain CIP 108541 and *Mycobacterium abscessus subsp bolletii* CIP 108297 (former *M. massiliense* reference strain) were sequenced at the Genopole of the Institut Pasteur by using the Genome Analyzer IIx (Illumina Inc., San Diego, USA) with a coverage rate of 175X and 170X, respectively. 36 bp single-end reads were generated and aligned against the reference genome of *M. abscessus* (EMBL accession number: CU458896) [41] by using MAQ [71]. In order to prevent the presence of potential amplification contaminants, duplicated reads were removed from the alignment maps. Two reads were considered as a duplicate if they shared the same mapping position, stemmed from the same DNA strand and possessed exactly the same sequence. In the case of duplicates, the read having the best quality sum was preserved. The resulting alignment maps were then analysed by using SNIFER (https://bitbucket.org/clafooty/tango/wiki/Home) for the SNP calling, which is based on a comparison of aligned read sequences to the reference genome from mapping positions. Mismatches detected were then filtered according to 5 stringent criteria: (i) a coverage sum > 10; (ii) a substitution frequency of at least 0.89; (iii) a mean quality of mapped bases > 20 according to the Sanger format; and both mean (iv) coverage and (v) quality >20 for the 10 bases surrounding the variant (−5/+5). So as to investigate large insertion-deletion events, each short-read data set was *de novo* assembled using the perl script VelvetOptimiser, provided with the VELVET package [72].

*Mycobacterium abscessus subsp. bolletii* CIP 108541 genomic sequence was deposited on NCBI whole genome shotgun project with accession number JRMF00000000. *Mycobacterium abscessus subsp. bolletii* CIP 108297 was deposited on NCBI whole genome shotgun project with accession number JRMG00000000.

### Mosaic strains DNA sequencing and genome assembly

Based on the results obtained from the Bayesian algorithm STRUCTURE, three “hybrid” strains from the MAB were selected for whole-genome sequencing (Strain M139, strain 23 and strain 137). Strain M139 came from a sputum sample from a Malaysian patient with a MAB lung infection; contigs were already generated and assembled in a former study [50]. Strains 137 and 23 (respectively *Mycobacterium abscessus subsp. bolletii* 137 and *Mycobacterium abscessus* 23) were taken from our laboratory collection. Libraries were constructed using the Nextera Kit (Illumina) from 50 ng of DNA according to Illumina’s recommendations. Pooled libraries were sequenced on an Illumina HiSeq-2000 platform to generate 100 bp paired reads, with the TruSeq PE Cluster kit v3 and TruSeq SBS kit v3 (Illumina). All reads were pre-processed to remove low quality or artefactual nucleotides. First, all nucleotides occurring at 5′ and 3′ ends and supported by a Phred quality score < 28 were trimmed off using SICKLE (https://github.com/najoshi/sickle). Second, contaminant oligonucleotides (i.e., library adaptors) were detected and trimmed off using ALIENTRIMMER [73]. Third, reads shorter than 45 nt after the aforementioned cleaning steps were discarded, as were those containing more than 5 % nucleotides with Phred score < 28. Finally, the program FQDUPLICATE (ftp://ftp.pasteur.fr/pub/gensoft/projects/fqtools) was used to discard every duplicate single- or paired-ends reads. A *de novo* assembly of the remaining reads was built with CLC Genomics Workbench version 3 (CLC Bio, Cambridge, MA). Contigs were then reordered using the MUMMER software.

The contigs of the three “hybrid” strains were compared with the three reference strains previously assembled (*M. abscessus* subspecies, *M. massiliense* subspecies, and *M. bolletii* subspecies) using NUCMER [74] and the delta-filter command (90 % minimum identity threshold on at least 400 nucleotides). The Show-Tilling script was then used to determine the order of the contigs (minimum 10 % coverage, maximal gap length 100,000 bp). The rejected contigs were manually checked and reintegrated in the final assembly if they had at least 96 % identity on > 1500 bp regions. The assembled genomes of strains M139, 23 and 137 are very similar to the reference strains (4,916,028 bp, 4,834,006 bp, and 5,011,043 bp, respectively), and represent at least 95 % of the size of the longest reference *M. abscessus subsp. abscessus* strain chromosome, that was sequenced by the Sanger method [41]. *Mycobacterium abscessus* 23 genomic sequence was deposited on NCBI whole genome shotgun project with accession number JRMD00000000. *Mycobacterium abscessus subsp. bolletii* 137 was deposited on NCBI whole genome shotgun project with accession number JRME00000000.

Additionally, the 6 analysed genomes were uploaded on the Genoscope MAGE database. Core and accessory genomes were identified using a Bidirectionnal Best Hit Method, with 50 % protein identity threshold. Manual gene annotation was performed for the genetic loci of interest using global information given by this platform (Interproscan domains, SwissProt similarities, FigFam) for each gene. All regions that were identified as putative genomic islands (strain specific genetic loci of more than 8 kb) were all blasted on the NCBI NR nucleotide database, and results showing more than 80 % identity on more than 75 % of the sequence were retrieved. Graphical representation of genomic loci of interest was performed using the GENOPLOTR Package [75], and alignments shown were performed on genetic loci of interest using ARTEMIS software [76] output file.

### Mobile elements detection

Mobile elements were searched in genome sequences. tRNA were identified using tRNA finder. Prophage elements were found using prophage finder (hit per prophage: 4; hit spacing: 3500). Putative insertion sequences were determined using IS finder (minimum score: 80), and manually checked with BLASTP on selected domains in order to verify the presence of recombinase or integrase genes. We checked for conjugation-associated genes in the 6 genomes (including aligned and non-aligned contigs) using traB/ftsK and virB/virD motifs on the MAGE platform (Interproscan domains, SwissProt similarities, FigFam). ESX genomic regions were identified by using a pBLAST search on all 6 reported full genomes with the *M. tuberculosis* ESX system core proteins EccCa (Rv3870), EccD (Rv3877) and MycP1 (Rv3883c). Type IV secretion/conjugation systems were annotated using CONJSCAN - T4SSSCAN tools [77].

## Additional files

Additional file 1: Figure S1. Plots of transitions (blue crosses) and transversions (green triangles) versus genetic distance. General time reversible (GTR) genetic distance for the eight concatenated gene fragments is plotted against the percent number of substitutions at all codon positions. S: transitions; V: transversions. No apparent saturation can be detected. (PDF 303 kb) Additional file 2: Figure S2. Likelihood Mapping Analysis for the full *Mycobacterium abscessus* complex data set (N = 280 strains; 8 concatenated genes). Phylogenetic noise was calculated using likelihood mapping analysis by analysing 10,000 random quartets. Each dot represents the likelihood of three possible tree topologies for each quartet. The dots localized close to the triangle vertices represent tree-like phylogenetic signal. Those in the centre and on the sides represent star-like and network-like signal, respectively. (PDF 85 kb) Additional file 3: Figure S3. Phylogenetic diversity of the *M. abscessus* complex (STs). The Neighbor-net tree was constructed by using concatenated sequences of the seven housekeeping genes used in the MLST scheme. This graph was constructed by using Splitstree version 4.13.1. Distances were estimated by using logdet distances. (PDF 264 kb) Additional file 4: Figure S4. A. Ms_tree_ representing the isolates characterization based on *rpoB* typing. B. Rates of false identification according to *rpoB* sequencing. Colored histograms correspond to assignments confirmed by the Bayesian algorithm (MLST based), whereas black fractions correspond to conflicting identifications (failure rate). (PDF 184 kb) Additional file 5: Table S1. List of the MAB isolates used for the clinical investigations. SST: Skin and soft tissue. CF: cystic fibrosis. HIV: human immunodeficiency virus. HCV: hepatitis C virus. COPD: chronic obstructive pulmonary disease. TB: tuberculosis. Lung infection: MAB isolate with NTM lung disease associated symptom following ATS criteria. Lung colonisation: MAB isolate without any NTM lung disease associated symptom following ATS criteria. (DOCX 37 kb) Additional file 6: Figure S5. Comparison among three *M. abscessus* sub-species. The Venn diagram shows the number of genes in each MAB sub-species type strain. Number of homologous genes (more than 50 % protein sequence identity using BBH method) are indicated at the intersections of the circles. (PDF 350 kb) Additional file 7: Figure S6.
*M. abscessus* sub-species-specific SNP whole genome density map. A: *M. massiliense* strain M139. B: *M. abscessus* strain 23. C: *M. massiliense* strain 137. Blue: *M. massiliense* specific SNPs; red: *M. abscessus* specific SNPs; yellow: *M. bolletii* specific SNPs. (PDF 1879 kb) Additional file 8: Figure S7. Remarkable features of *M. massiliense* strain M139 genome. A Genomic exchanges at *erm(41)* locus. *M. massiliense* strain M139 Contig 22. Subspecies identity is indicated (blue: *M. massiliense*, red: *M. abscessus*, yellow: *M. bolletii*, grey: no significant subspecies attribution). *Erm(41)* (purple). B: non-aligned contig of *M. massiliense* strain M139 containing an ESX locus similar to *M. marinum* p-RAW conjugative plasmid (light blue: typeVII/ESX and type IV coding genes). (PDF 35 kb) Additional file 9: Figure S8. Phylogenetic reconstructions of the MAB sequence types, for each of the 7 housekeeping genes used for MLST study. Phylogenetic trees were made using BioNJ method (Gascuel O. An improved version of NJ algorithm based on a simple model of sequence Data. Mol Biol Evol. 1997;14:685–695.) with 500 replicate, and Kimura 2 parameters correction. (PDF 72 kb) Additional file 10: Figure S9. Non-synonymous *vs* synonymous mutations in *Mycobacterium abscessus* gene fragments used for MLST studies. (PDF 74 kb)
