# Supplementary figures and images for: Genome-wide mosaicism within Mycobacterium abscessus: evolutionary and epidemiological implications
Source: BMC Genomics. 2016 Feb 17;17:118. doi: 10.1186/s12864-016-2448-1 (PMC4756508; doi:10.1186/s12864-016-2448-1)

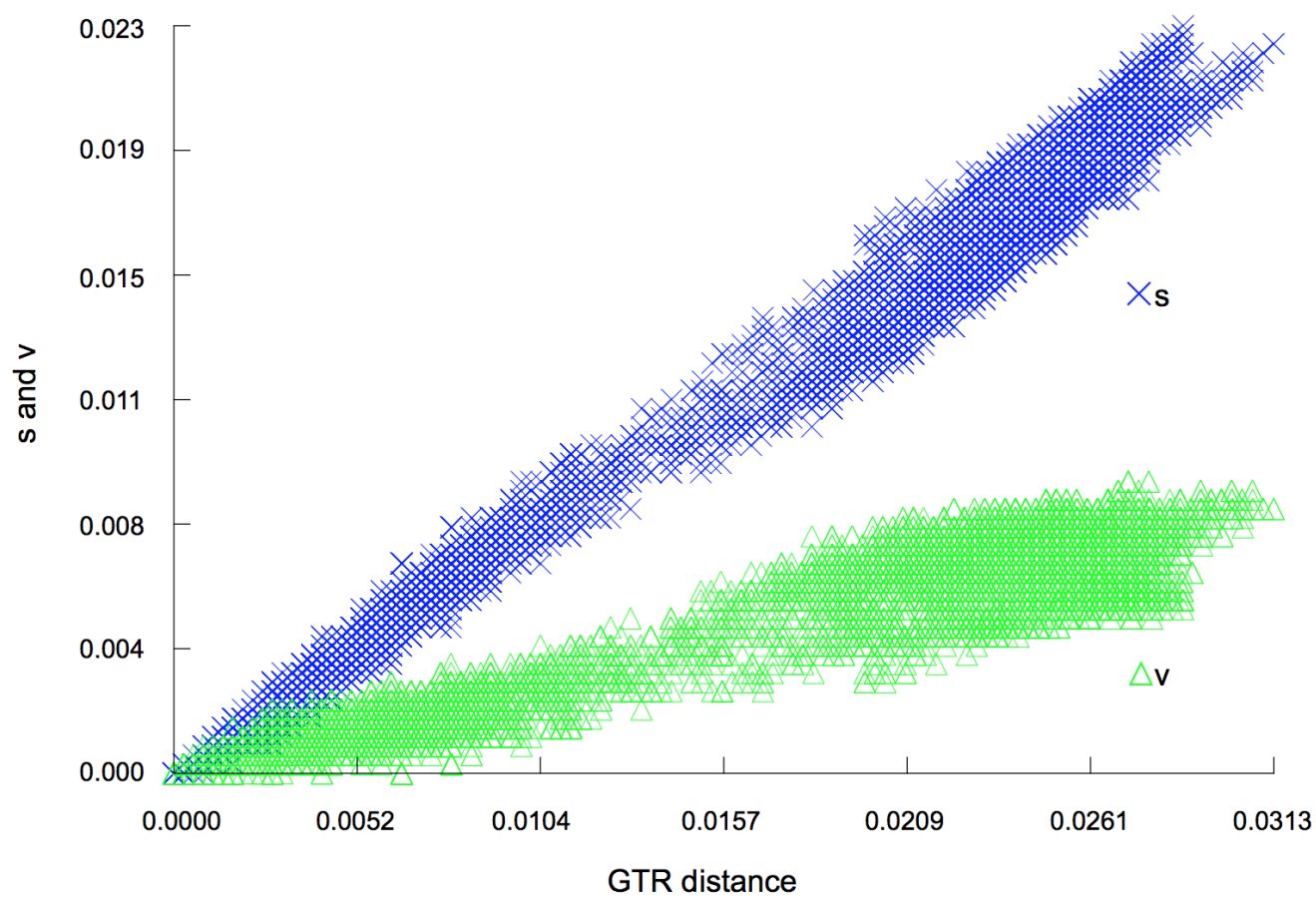

Figure S1

Supplement: Additional file 1: Figure S1. — Plots of transitions (blue crosses) and transversions (green triangles) versus genetic distance. General time reversible (GTR) genetic distance for the eight concatenated gene fragments is plotted against the percent number of substitutions at all codon positions. S: transitions; V: transversions. No apparent saturation can be detected. (PDF 303 kb) [file 12864_2016_2448_MOESM1_ESM.pdf]

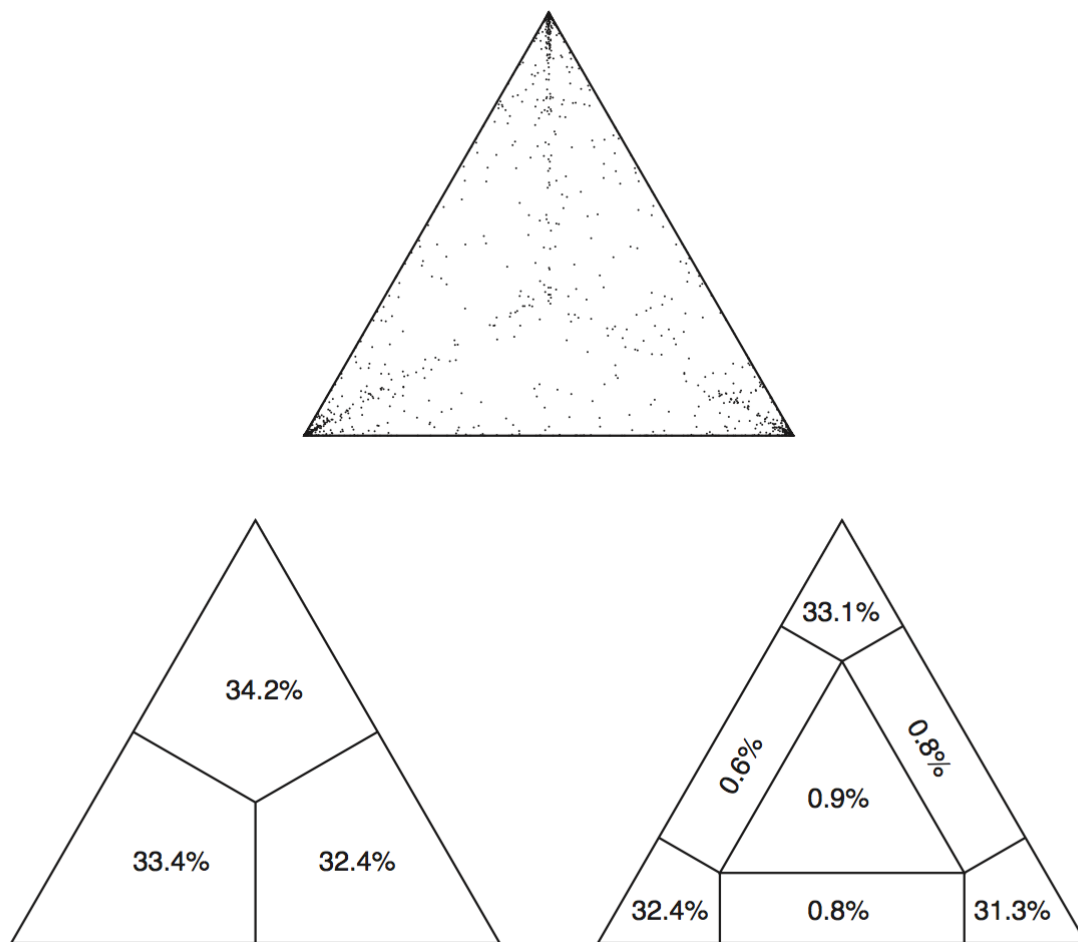

Figure S2

Supplement: Additional file 2: Figure S2. — Likelihood Mapping Analysis for the full Mycobacterium abscessus complex data set (N = 280 strains; 8 concatenated genes). Phylogenetic noise was calculated using likelihood mapping analysis by analysing 10,000 random quartets. Each dot represents the likelihood of three possible tree topologies for each quartet. The dots localized close to the triangle vertices represent tree-like phylogenetic signal. Those in the centre and on the sides represent star-like and network-like signal, respectively. (PDF 85 kb) [file 12864_2016_2448_MOESM2_ESM.pdf]

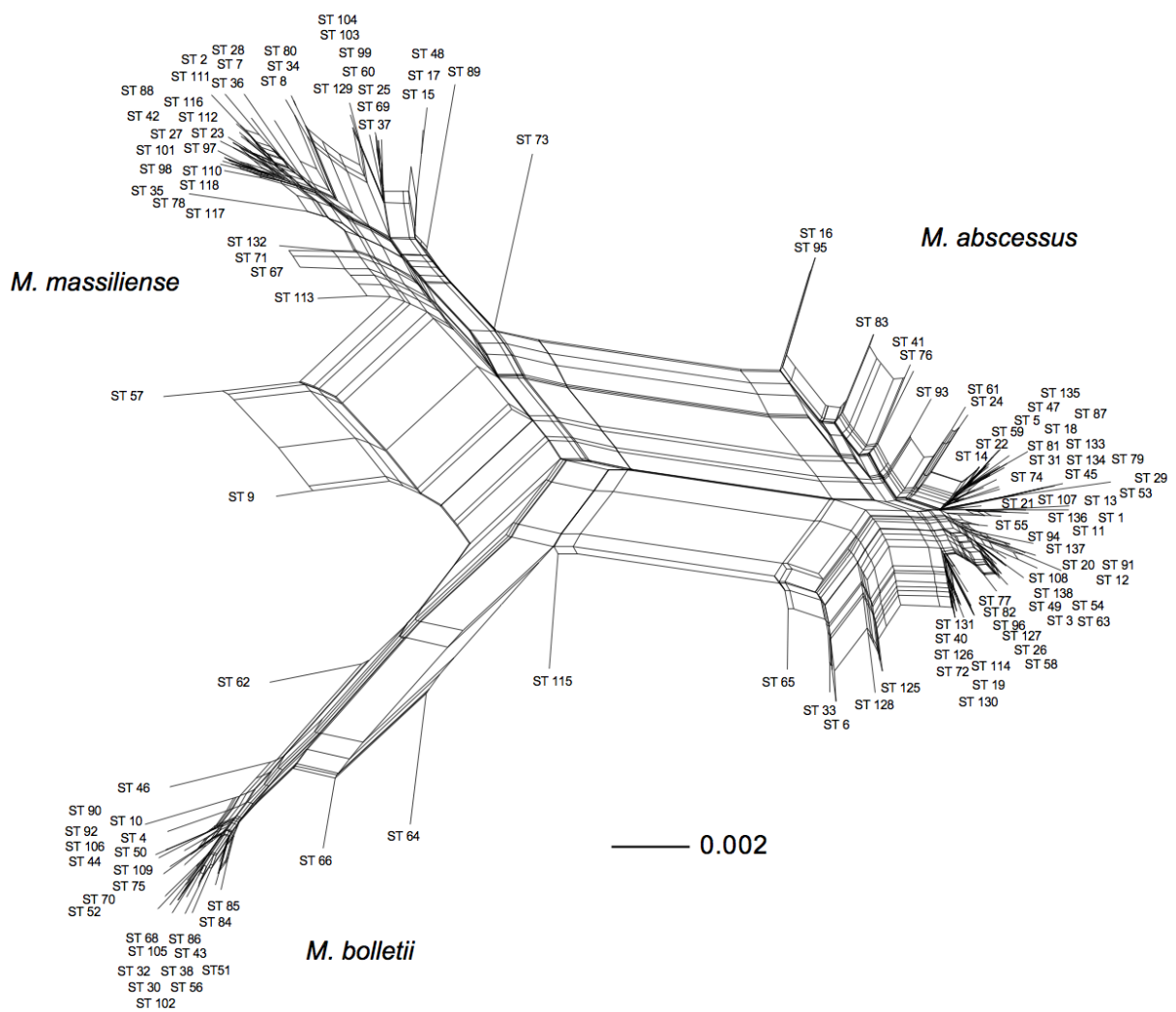

Figure S3

Supplement: Additional file 3: Figure S3. — Phylogenetic diversity of the M. abscessus complex (STs). The Neighbor-net tree was constructed by using concatenated sequences of the seven housekeeping genes used in the MLST scheme. This graph was constructed by using Splitstree version 4.13.1. Distances were estimated by using logdet distances. (PDF 264 kb) [file 12864_2016_2448_MOESM3_ESM.pdf]

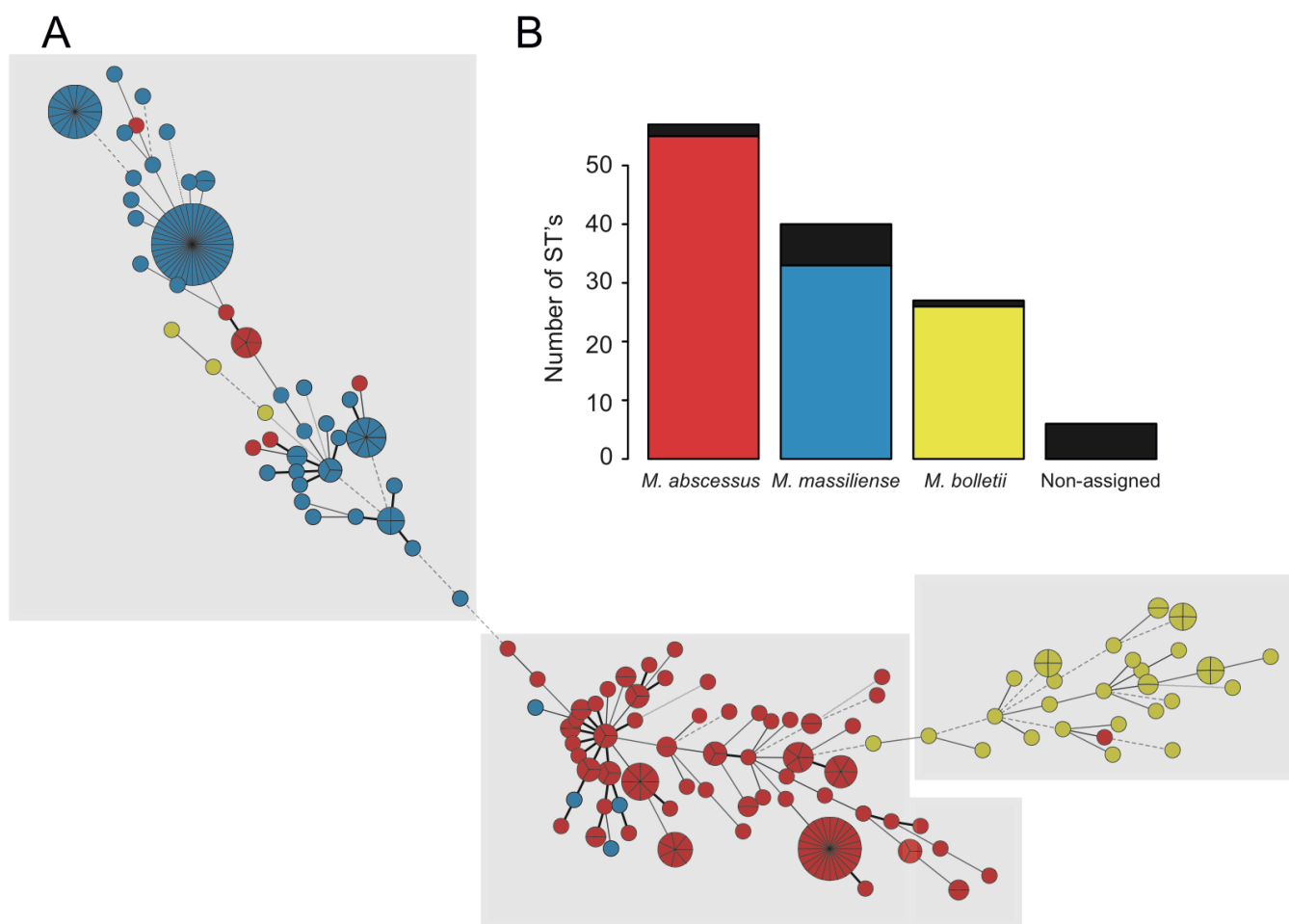

Figure S4

Supplement: Additional file 4: Figure S4. — A. Mstree representing the isolates characterization based on rpoB typing. B. Rates of false identification according to rpoB sequencing. Colored histograms correspond to assignments confirmed by the Bayesian algorithm (MLST based), whereas black fractions correspond to conflicting identifications (failure rate). (PDF 184 kb) [file 12864_2016_2448_MOESM4_ESM.pdf]

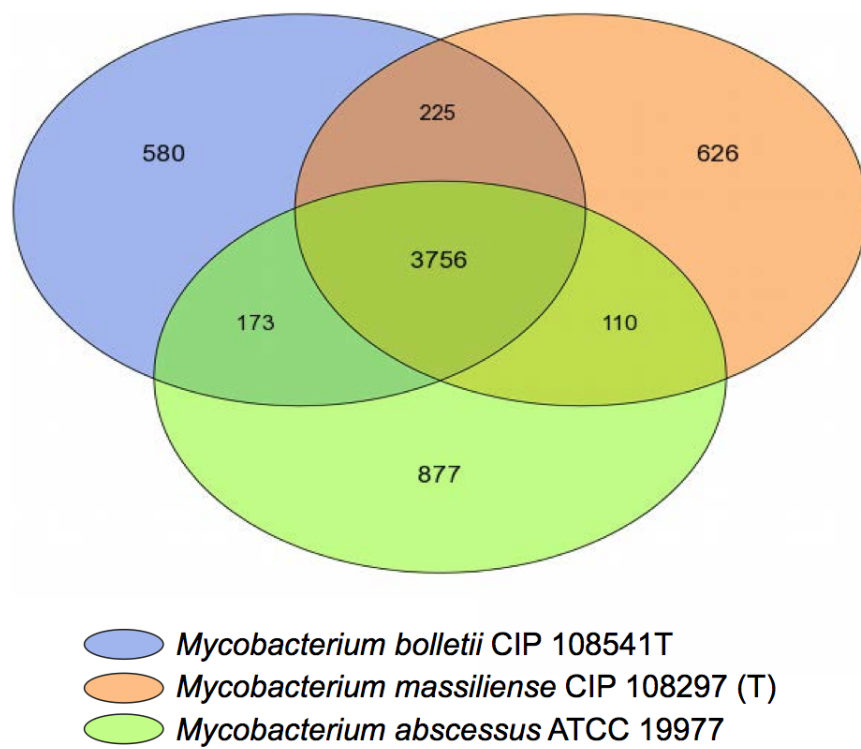

Figure S5

Supplement: Additional file 6: Figure S5. — Comparison among three M. abscessus sub-species. The Venn diagram shows the number of genes in each MAB sub-species type strain. Number of homologous genes (more than 50 % protein sequence identity using BBH method) are indicated at the intersections of the circles. (PDF 350 kb) [file 12864_2016_2448_MOESM6_ESM.pdf]

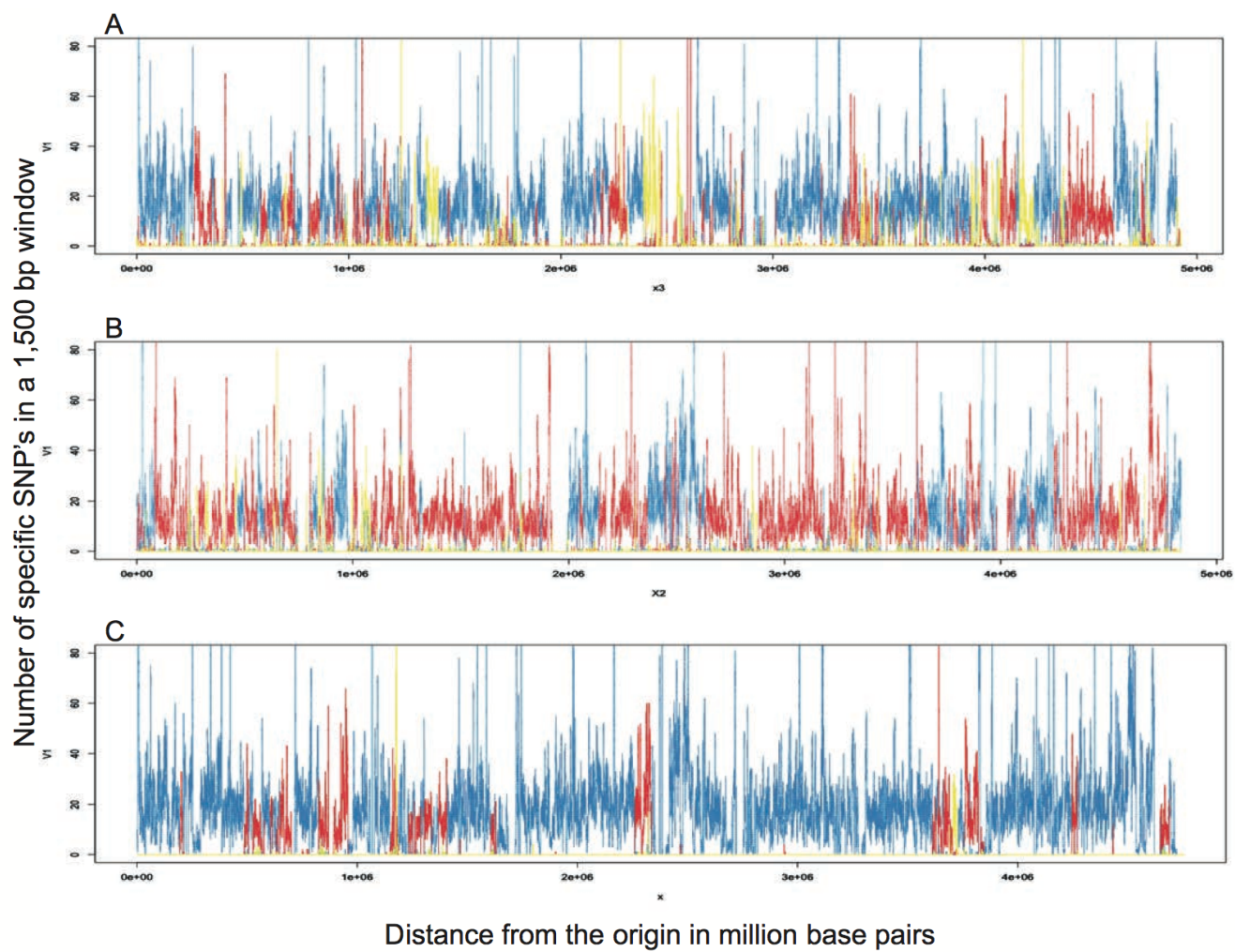

Figure S6

Supplement: Additional file 7: Figure S6. — M. abscessus sub-species-specific SNP whole genome density map. A: M. massiliense strain M139. B: M. abscessus strain 23. C: M. massiliense strain 137. Blue: M. massiliense specific SNPs; red: M. abscessus specific SNPs; yellow: M. bolletii specific SNPs. (PDF 1879 kb) [file 12864_2016_2448_MOESM7_ESM.pdf]

Figure S7: remarkable genomic features of *M. massiliense* strain M139

A

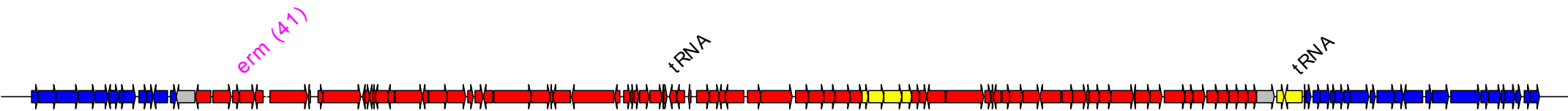

B

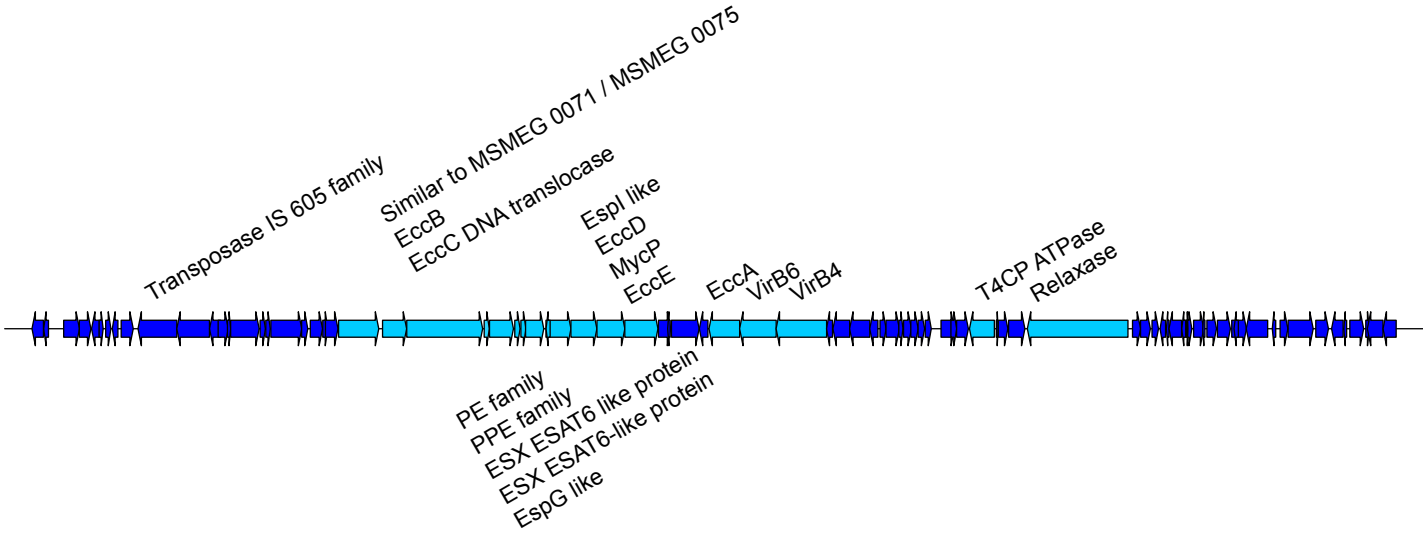

20 kb

Supplement: Additional file 8: Figure S7. — Remarkable features of M. massiliense strain M139 genome. A Genomic exchanges at erm(41) locus. M. massiliense strain M139 Contig 22. Subspecies identity is indicated (blue: M. massiliense, red: M. abscessus, yellow: M. bolletii, grey: no significant subspecies attribution). Erm(41) (purple). B: non-aligned contig of M. massiliense strain M139 containing an ESX locus similar to M. marinum p-RAW conjugative plasmid (light blue: typeVII/ESX and type IV coding genes). (PDF 35 kb) [file 12864_2016_2448_MOESM8_ESM.pdf]

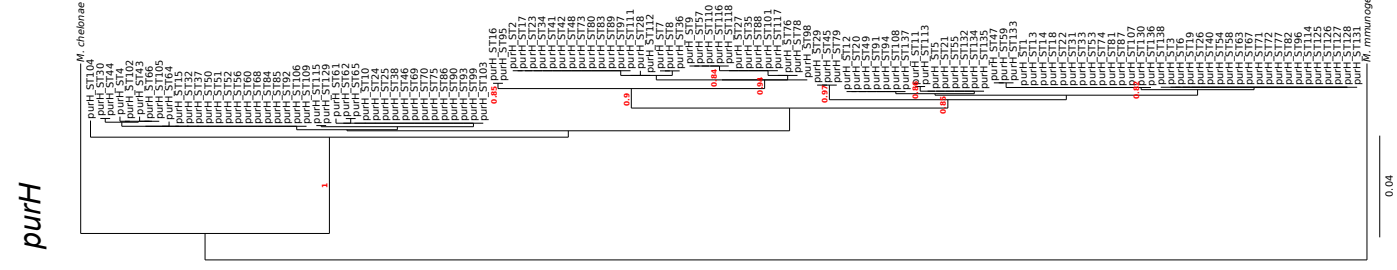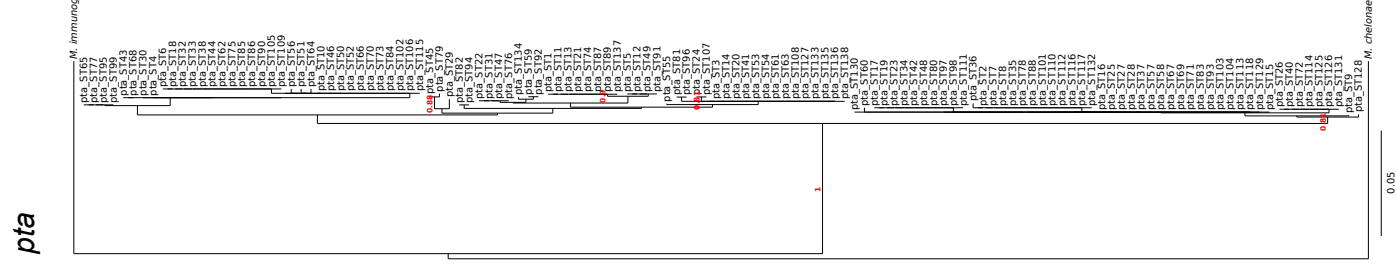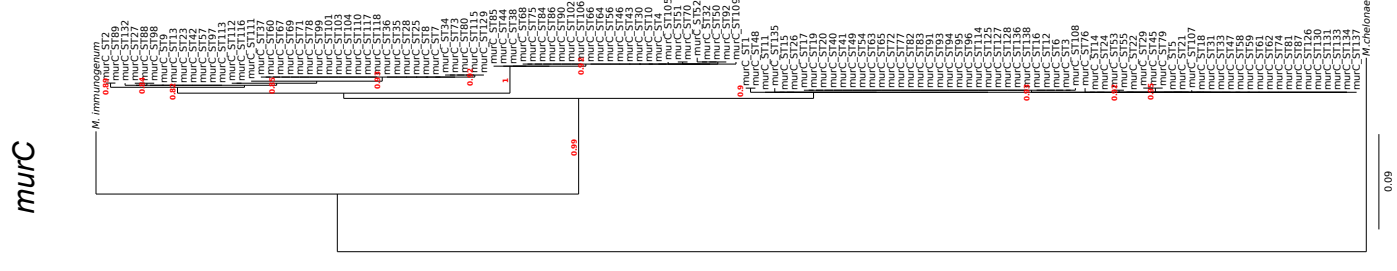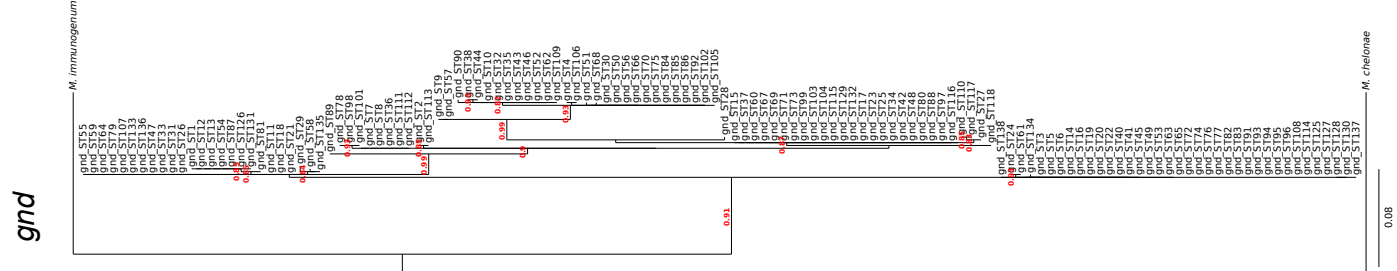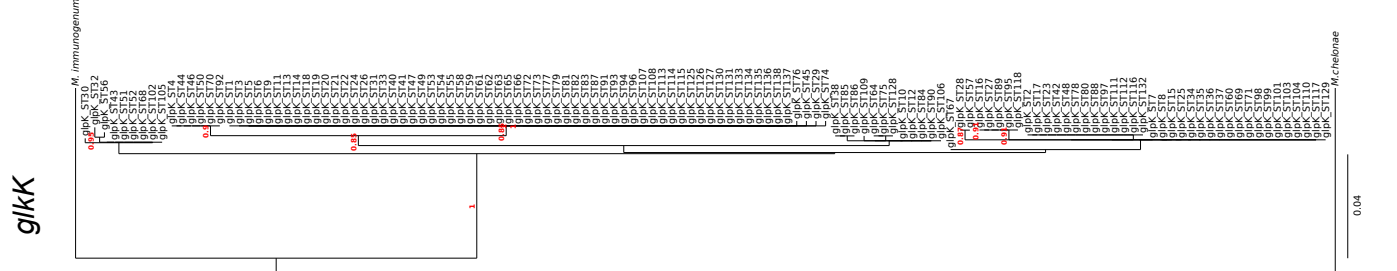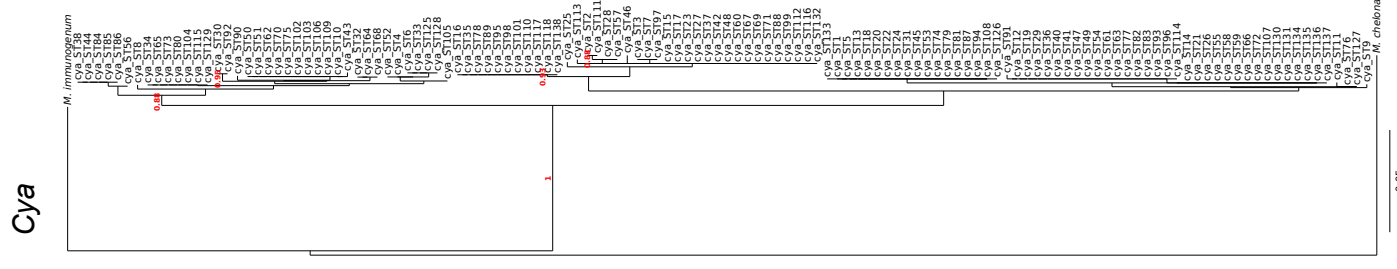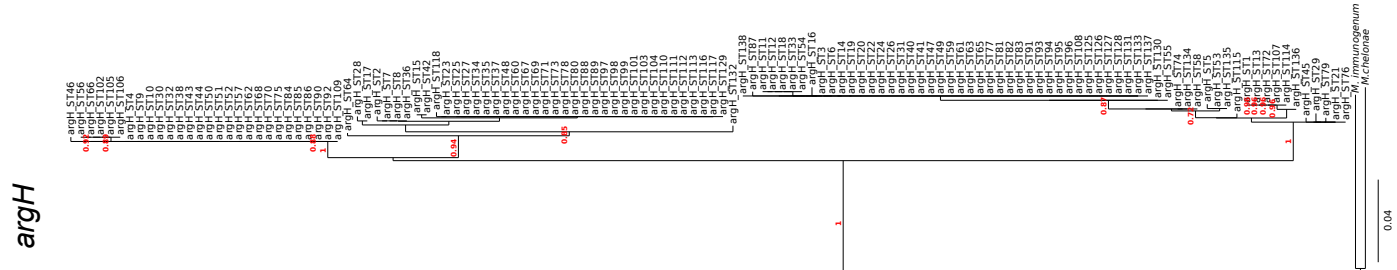

## Figure S8

Supplement: Additional file 9: Figure S8. — Phylogenetic reconstructions of the MAB sequence types, for each of the 7 housekeeping genes used for MLST study. Phylogenetic trees were made using BioNJ method (Gascuel O. An improved version of NJ algorithm based on a simple model of sequence Data. Mol Biol Evol. 1997;14:685–695.) with 500 replicate, and Kimura 2 parameters correction. (PDF 72 kb) [file 12864_2016_2448_MOESM9_ESM.pdf]

Figure S9

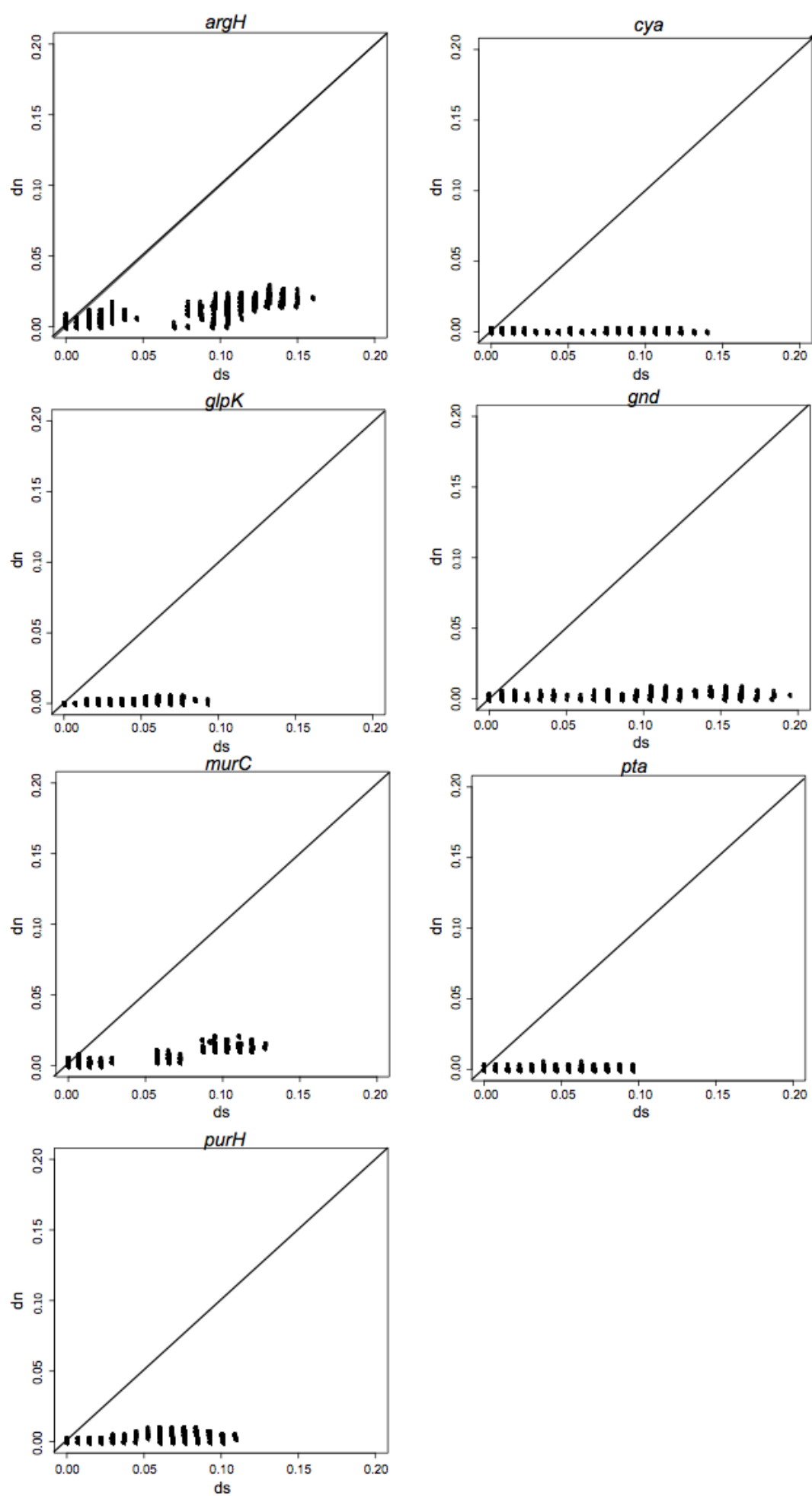

Supplement: Additional file 10: Figure S9. — Non-synonymous vs synonymous mutations in Mycobacterium abscessus gene fragments used for MLST studies. (PDF 74 kb) [file 12864_2016_2448_MOESM10_ESM.pdf]
