# Supplementary material for: Genome-wide mosaicism within Mycobacterium abscessus: evolutionary and epidemiological implications
Source: BMC Genomics. 2016 Feb 17;17:118. doi: 10.1186/s12864-016-2448-1 (PMC4756508; doi:10.1186/s12864-016-2448-1)
Supplement: Additional file 5: Table S1. — List of the MAB isolates used for the clinical investigations. SST: Skin and soft tissue. CF: cystic fibrosis. HIV: human immunodeficiency virus. HCV: hepatitis C virus. COPD: chronic obstructive pulmonary disease. TB: tuberculosis. Lung infection: MAB isolate with NTM lung disease associated symptom following ATS criteria. Lung colonisation: MAB isolate without any NTM lung disease associated symptom following ATS criteria. (DOCX 37 kb) [file 12864_2016_2448_MOESM5_ESM.docx]

| strain | ST | year | location | sample type | disease | underlying disease |
| --- | --- | --- | --- | --- | --- | --- |
| 1 | 1 | 1953 | USA | articular liquid | knee abscess infection | no |
| 2 | 2 | 2001 | France | respiratory | lung infection | CF |
| 8 | 4 | 2005 | France | respiratory | lung infection | CF |
| 10 | 2 | 2004 | France | respiratory | lung colonisation | CF |
| 12 | 5 | 2004 | France | respiratory | lung colonisation | CF |
| 14 | 6 | 2004 | France | respiratory | lung infection | CF |
| 16 | 2 | 2000 | France | respiratory | lung colonisation | CF |
| 18 | 8 | 2004 | France | respiratory | lung colonisation | CF |
| 19 | 9 | 2005 | France | respiratory | lung colonisation | CF |
| 20 | 43 | 2004 | France | respiratory | lung colonisation | CF |
| 22 | 11 | 2005 | France | respiratory | lung infection | CF |
| 23 | 20 | 2005 | France | respiratory | lung colonisation | CF |
| 26 | 12 | 2004 | France | respiratory | lung infection | CF |
| 28 | 33 | 2004 | France | respiratory | lungcolonisation | CF |
| 34 | 21 | 2002 | France | respiratory | lung infection | CF |
| 35 | 22 | 2005 | France | respiratory | lung infection | CF |
| 38 | 44 | 2005 | France | respiratory | lung colonisation | CF |
| 39 | 1 | 2004 | France | respiratory | Lung colonisation | CF |
| 41 | 24 | 2004 | France | respiratory | Lung colonisation | CF |
| 45 | 23 | 2000 | France | respiratory | lung infection | CF |
| 46 | 26 | 2005 | France | respiratory | lung infection | CF |
| 47 | 28 | 2004 | France | respiratory | Lung colonisation | CF |
| 49 | 23 | 2005 | France | respiratory | Lung colonisation | CF |
| 54 | 1 | 2005 | France | respiratory | lung infection | CF |
| 55 | 31 | 2005 | France | respiratory | Lung colonisation | CF |
| 59 | 33 | 2001 | France | respiratory | lung infection | CF |
| 62 | 1 | 2004 | France | respiratory | Lung colonisation | CF |
| 63 | 34 | 2004 | France | respiratory | lung infection | CF |
| 65 | 10 | 2002 | France | respiratory | lung infection | CF |
| 71 | 37 | 2004 | France | respiratory | Pneumonia | Addisons Disease |
| 92 | 18 | 2006 | France | respiratory | lung infection | CF |
| 95 | 18 | 2007 | France | pericardial puncture | pericarditis | not known |
| 119 | 52 | 2008 | France | respiratory | Pneumonia | HIV |
| 120 | 49 | 2008 | France | SST | SST infection | Tattooing |
| 121 | 49 | 2008 | France | SST | SST infection | post-surgery |
| 122 | 61 | 2004 | France | respiratory | lung infection | CF |
| 126 | 64 | 2004 | France | respiratory | lung infection | CF |
| 128 | 41 | 2005 | France | respiratory | lung infection | CF |
| 129 | 23 | 2006 | Brazil | SST | SST infection | unknown |
| 160 | 23 | 2007 | France | respiratory | Pneumonia | kidney transplantation |
| 161 | 34 | 2006 | France | respiratory | Pneumonia | COPD |
| 162 | 37 | 2007 | France | respiratory | Pneumonia | Ondines curse |
| 163 | 1 | 2007 | France | respiratory | Pneumonia | Pulmonary fibrosis |
| 164 | 2 | 2006 | France | respiratory | Pneumonia | Asthma |
| 165 | 80 | 2006 | France | respiratory | Pneumonia | COPD |
| 166 | 72 | 2007 | France | lymph node | Adenitis | HIV, Hodgkins disease |
| 167 | 76 | 2007 | France | respiratory | Pneumonia | Wegeners disease |
| 169 | 81 | 2008 | France | SST | SST infection | unknown |
| 170 | 2 | 2008 | France | respiratory | Pneumonia | COPD |
| 171 | 82 | 2007 | France | respiratory | Pneumonia | Previous tuberculosis |
| 172 | 73 | 2008 | France | respiratory | Pneumonia | HCV infection |
| 174 | 33 | 2007 | France | respiratory | Pneumonia | COPD |
| 176 | 1 | 2007 | France | respiratory | Pneumonia | COPD |
| 177 | 78 | 2008 | France | respiratory | Pneumonia | COPD |
| 178 | 2 | 2007 | France | respiratory | Pneumonia | COPD |
| 179 | 83 | 2008 | France | respiratory | Bronchitis | lung cancer |
| 180 | 86 | 2006 | France | respiratory | Pneumonia | HIV |
| 181 | 1 | 2006 | France | respiratory | Pneumonia | Lymphoma |
| 182 | 84 | 2007 | France | articular liquid | Knee arthritis | unknown |
| 184 | 74 | 2007 | France | respiratory | Pneumonia | Previous TB |
| 186 | 79 | 2006 | France | respiratory | Pneumonia | Lung transplantation |
| 187 | 23 | 2008 | France | respiratory | Pneumonia | COPD |
| 188 | 7 | 2008 | France | SST | SST infection | Trauma |
| 189 | 87 | 2008 | France | articular puncture | Arthritis | unknown |
| 190 | 49 | 2008 | France | respiratory | Pneumonia | COPD |
| 191 | 79 | 2008 | France | respiratory | Pneumonia | Diabetes |
| 192 | 88 | 2006 | France | respiratory | Pneumonia | COPD |
| 193 | 1 | 2008 | France | respiratory | Pneumonia | Previous TB |
| 201 | 85 | 2009 | France | respiratory | Pneumonia | Breast cancer |
| 216 | 2 | 1999 | France | respiratory | lung infection | CF |
| 218 | 19 | 2004 | France | respiratory | Lung colonisation | CF |
| 219 | 33 | 1998 | France | respiratory | lung infection | CF |
| 223 | 96 | 1999 | France | respiratory | Lung colonisation | CF |
| 232 | 23 | 2002 | Malaysia | lymph node | Cervical lymphadernitis | unknown |
| 233 | 109 | 2002 | Malaysia | respiratory | lung infection | no |
| 241 | 55 | 2010 | Malaysia | respiratory | lung infection | no |
| 242 | 49 | 2010 | Malaysia | respiratory | lung infection | Lung cancer COPD |
| 243 | 23 | 2010 | Malaysia | respiratory | lung infection | TB |
| 244 | 112 | 2010 | Malaysia | respiratory | lung infection | COPD and TB |
| 245 | 23 | 2010 | Malaysia | respiratory | lung infection | breast cancer |
| 246 | 113 | 2010 | Malaysia | respiratory | lung infection | asthma |
| 247 | 1 | 2010 | Malaysia | respiratory | lung infection | no |
| 248 | 23 | 2010 | Malaysia | respiratory | lung infection | no |
| 249 | 115 | 2010 | Malaysia | respiratory | Lung infection | no |
| 250 | 116 | 2010 | Malaysia | respiratory | lung infection | no |
| 251 | 23 | 2010 | Malaysia | respiratory | lung infection | no |
| 252 | 117 | 2010 | Malaysia | respiratory | lung infection | previous TB |
| 253 | 114 | 2010 | Malaysia | respiratory | lung infection | TB |
| 254 | 63 | 2010 | Malaysia | respiratory | lung infection | no |
| 255 | 117 | 2010 | Malaysia | respiratory | lung infection | no |
| 256 | 117 | 2010 | Malaysia | respiratory | lung infection | TB with haemoptysis and bronchiectasis |
| 257 | 2 | 2010 | Malaysia | respiratory | lung infection | prostate cancer |
| 258 | 48 | 2010 | Malaysia | respiratory | lung infection | no |
| 259 | 117 | 2010 | Malaysia | respiratory | lung infection | TB |
| 260 | 118 | 2010 | Malaysia | respiratory | lung infection | mixed infection M. fortuitum |
| 262 | 128 | 2011 | Taiwan | respiratory | lung infection | Diabetes, Dyslipidemia, COPD, Bronchiectasis |
| 265 | 135 | 2011 | Taiwan | respiratory | lung infection | Previous pulmonary TB, chronic hepatitis C |
| 266 | 136 | 2011 | Taiwan | respiratory | lung infection | previous pulmonary TB, bronchiectasis, COPD |
| 267 | 125 | 2011 | Taiwan | respiratory | lung infection | Congestive heart failure, chronic kidney disease |
| 269 | 127 | 2011 | Taiwan | respiratory | lung infection | T cell lymphoma |
| 274 | 137 | 2011 | Taiwan | peritoneum | Peritonitis | End stage renal disease, hypertension, diabetes |
| 275 | 138 | 2011 | Taiwan | respiratory | lung infection | Bronchiectasis, chronic hepatitis C, hypertension |
